# Chemical inhibition of α-mannosidase in *S. mansoni* schistosomula leads to reshaped N-glycosylation associated with developmental alterations

**DOI:** 10.3389/fpara.2026.1930872

**Published:** 2026-09-15

**Authors:** Eleonore B. Kuhlemaijer, Tom Veldhuizen, Quirine C. A. Hoekstra, Jinthe A. Umbach, Mathijs J. Niezen, Josephine E. Forde-Thomas, Gabriel Rinaldi, Karl F. Hoffmann, Angela van Diepen, Cornelis H. Hokke

**Affiliations:** 1 Leiden University Center for Infectious Diseases – Research (LUCID-R), Leiden University Medical Center, Leiden, Netherlands; 2 Department of Life Sciences (DLS), Aberystwyth University, Aberystwyth, United Kingdom; 3 Department of Biology, University of Oxford, Oxford, United Kingdom

**Keywords:** N-glycans, parasite development, parasite glycobiology, *Schistosoma mansoni*, schistosomula, α-mannosidase inhibition

## Abstract

Schistosomes are parasitic flatworms that infect over 230 million people worldwide. As continuous exposure and high-reinfection rates cause severe disease in inhabitants of endemic areas, there is an urgent need for prophylactic vaccines or drugs that induce protective immunity. N-glycosylation is a conserved process in eukaryotes, including schistosomes, that affects various cellular and developmental biological processes. Schistosomes display characteristic developmental N-glycans that are important in parasite-host interaction and intra-mammalian development, which is associated with a changing N-glycan profile. In this study, we investigate whether application of the α-mannosidase inhibitors kifunensine and swainsonine during long-term *in vitro* culture of *S. mansoni* schistosomula leads to altered N-glycosylation and phenotypic changes in the parasite’s development. We first analysed the effect of the inhibitors on schistosomula motility and phenotype, and showed that there was no overt toxicity linked to these compounds. Next, we examined the schistosomula N-glycosylation profiles during six weeks of culture. Within the first week (W1), the N-glycosylation changed dynamically in the schistosomula and exposure to inhibitors led to distinct profiles compared to the control. Control parasites were marked by oligomannose (30%) and complex (60%) N-glycans, kifunensine treatment led to a high proportion of oligomannose N-glycans (72%), specifically M8 and M9, and swainsonine-treated schistosomula were characterised by oligomannose (44%), hybrid (11%) N-glycans and free N-glycans with one core-GlcNAc (16%) at W1 post-compound treatment. Continued treatment conserved the distinct profiles, while discontinuation of treatment after W1 caused the N-glycans of kifunensine and swainsonine schistosomula to return to a profile similar to controls at W3. Interestingly, schistosomula treated continuously with kifunensine developed local oedema from W4 onwards, which was most profound at W6. However, discontinued treatment after W1 did not lead to a visible phenotype at W4 or later. In conclusion, prolonged alterations in schistosomula N-glycosylation led to disturbed development whereas short term glycan changes during the first week only, did not affect the parasite’s normal development. Our data indicate that glyco-remodelled live schistosomes provide an attractive approach to study the contribution of glycans in intra-mammalian parasite development and survival.

## Introduction

Schistosomes are multicellular eukaryotic parasites that cause schistosomiasis, a neglected tropical disease (Hotez et al., 2014) with more than 230 million people infected worldwide (Vos et al., 2012). The parasite has a complex life cycle that includes both an intermediate host and a definitive host, being (sub)tropical freshwater snails and mammals, respectively. Development of sterile immunity against schistosomes is rare (Colley and Secor, 2014), and adult parasites can survive for decades inside the human host (Staehelin et al., 2025). An important contributor in parasite survival is its ability to evade and modulate host immunity (Angeles et al., 2020). In previous studies, schistosome-derived glycans have been shown to steer cellular immune responses (Faveeuw et al., 2003; Everts et al., 2012; Meevissen et al., 2012; Kuipers et al., 2020) or elicit antibody production (Eberl et al., 2001; Naus et al., 2003; Nyame et al., 2003; Kariuki et al., 2008; Yang et al., 2019). Glycans are carbohydrates attached to proteins (N- or O-glycans) or lipids (glycosphingolipids or GSLs), adding an additional layer of complexity to these macromolecules (Varki and Kornfeld, 2022). Out of the three main glycan types, N-glycans are a conserved type that affect fundamental processes such as protein folding and receptor-ligand binding (Takahashi et al., 2016; Shin et al., 2025). Consequently, altered N-glycosylation, due to mutations, environmental, biological or experimental triggers, can cause strong biological effects such as those observed in congenital disorders of glycosylation (Ficicioglu and Stepien, 2001; Stanley, 2024), lysosomal storage disease (Cook et al., 2014) and altered immune function (Demetriou et al., 2001). N-glycosylation is a highly regulated enzymatic process that starts in the endoplasmic reticulum (ER), during and after protein translation (Ruiz-Canada et al., 2009), and proceeds in the Golgi. The initial phase is highly conserved within eukaryotes (Schwarz and Aebi, 2011) and consists of transfer of the Glc3Man9GlcNAc2 precursor to the growing peptide chain, followed by removal of Glc residues during the protein folding process, and specific removal of α1-2-linked mannose residues before further processing in the Golgi. As a result, fully processed N-linked glycans have a common core structure, consisting of Manα1-3(Manα1-6)Manβ1-4GlcNAcβ1–4GlcNAcβ1 (Stanley et al., 2022), complemented with distinctive motifs that are characteristic for a cell, state or species. For example, human N-glycans often contain terminal sialic acids, which have not been found in schistosomes (Hokke and Deelder, 2001; Smit et al., 2015). Conversely, core β1-2xylose as present on schistosome N-glycosylation is not observed in human N-glycans (Bardor et al., 2003; Smit et al., 2015).

Schistosomes have characteristic N-glycan motifs, including the structures LacdiNAc (LDN), (multi-)fucosylated LDN and LacNAc (LN) (Yoshino et al., 2012). Additionally, each schistosome species and life stage is characterised by its own N-glycosylation profile (Smit et al., 2015; Petralia et al., 2026). Inside the human host, schistosomes develop from larvae into adult worms over a period of five to seven weeks (Colley and Secor, 2014). During this time, the parasites encounter various environments and undergo major molecular and phenotypic changes (Wangwiwatsin et al., 2020). First, during penetration of the host’s skin, cercariae lose their tail and shed their glycocalyx within hours (Marikovsky et al., 1986; Hambrook and Hanington, 2021). After traversing the skin, schistosomula enter the circulation, where they migrate to the lungs and subsequently to the portal system in the liver. Ultimately, they reach the mesenteric (*Schistosoma* (*S.*) *mansoni* and *S. japonicum*) or perivesicular veins (*S. haematobium*) (Colley and Secor, 2014). During this journey, the schistosomula mature into female or male adult parasites, thereby increasing in size and cell number, forming pairs and becoming sexually mature (Clegg, 1965). Meanwhile, the N-glycan profile of these worms also changes. For instance, presence of N-glycans with core xyloses and fucosylated LN (Lewis X; LeX) decreases and LDN increases (Smit et al., 2015) and sex-specific N-glycans distinguish adult female from male worms (Wuhrer et al., 2006). Interestingly, N-glycans present on extracellular vesicles (EVs) released by the parasite change in a similar fashion (Kuipers et al., 2020, Kuipers et al., 2023). N-glycans on these EVs, and on other parasitic molecules too, mediate binding and uptake by host immune cells through recognition by C-type lectin receptors (Everts et al., 2012; Kuipers et al., 2020), illustrating the fundamental role of N-glycosylation in immune recognition and parasite-host interaction.

Recently, we generated *ex vivo* cultured adult *S. mansoni* schistosomes with altered N-glycosylation using two α-mannosidase inhibitors (Kuhlemaijer et al., 2026). Kifunensine is an inhibitor of α-mannosidase I (Elbein et al., 1990), known to increase the production of high mannose N-glycans (Morikawa et al., 2022), and swainsonine inhibits Golgi α-mannosidase II as well as lysosomal and cytosolic α-mannosidases (Tulsiani et al., 1982; Dorling et al., 1983), leading to production of hybrid N-glycans and free N-glycans with once core-GlcNAc (Morikawa et al., 2022). In accordance with previous studies, we found that kifunensine-treated parasites were dominated by oligomannoses, specifically with nine (M9) or eight (M8) mannoses, and swainsonine-treated schistosomes had increased M5, hybrid and free N-glycans (Elbein et al., 1990; Choi et al., 2018; Morikawa et al., 2022). However, we did not observe any negative effects on motility or morphology in the glyco-remodelled adult worms. Yet, because of the extensive development of the schistosomula, accompanied by the changing N-glycan profile (Smit et al., 2015), N-glycosylation could impact the larval development and concurrent interaction with the human host. To test the effect of perturbed N-glycosylation on schistosomula development, we cultured *S. mansoni* larvae *in vitro*, mimicking their maturation from cercariae to late stage schistosomula, and remodelled their N-glycosylation with kifunensine and swainsonine. The parasites were cultured from newly transformed schistosomula for up to six weeks, while we followed their N-glycosylation and development. Additionally, we investigated the effect of discontinuing the treatment with kifunensine and swainsonine after one week. In this study, we show that altered N-glycosylation can affect schistosome development *in vitro*, suggesting that glycan remodelling constitutes an interesting approach to study developmental biology of schistosomula and the possible identification of glycan-based putative drug or vaccine targets.

## Materials and methods

### Ethics statement

All procedures performed at Aberystwyth University (AU) on mice adhered to the United Kingdom Home Office Animals (Scientific Procedures) Act of 1986 (under project licence PP2955700) as well as the European Union Animals Directive 2010/63/EU and were approved by AU’s Animal Welfare and Ethical Review Bodies (AWERB). All procedures at Leiden University Medical Center (LUMC) on hamsters were performed in accordance with the Guide for the Care and Use of Laboratory Animals of the Institute for Laboratory Animal Research and have received approval from the Dutch ‘Central Authority for Scientific Procedures on Animals’ (CCD, License number: AVD11600202215904).

### Compound handling

Dimethyl sulfoxide (DMSO), auranofin and praziquantel were purchased from Sigma-Aldrich (Saint Louis, MO, USA). Each compound was dissolved in DMSO and stored at -20°C. Kifunensine (Cayman Chemical, Ann Arbor, MI, USA) and swainsonine (Biotechne, Minneapolis, MN, USA) were dissolved in PBS and stored at -20°C.

### Cercariae transformation

*S. mansoni* cercariae (Puerto Rican-strain) were obtained from infected *Biomphalaria glabrata* snails. Cercariae shedding was achieved through light exposure in Bar-le-Duc water (Bar-le-Duc, Utrecht, the Netherlands) at 30 °C for two hours or in Lepple water (Lewis, 1998) at 26 °C for 1.5 hours. Next, cercariae were kept on ice in the dark for one to two hours. Transformation of cercariae (Colley and Wikel, 1974) was performed at 4 °C as much as possible. Cercariae were centrifuged at 300 × *g* for 5 minutes (min) with minimal brake. The supernatant was discarded, replaced by cold Basal Medium Eagle (BME; Gibco, Paisly, UK) and cercariae were centrifuged at 300 × g for 3 min. This wash step was repeated twice, after which the cercariae were left in ~6 mL BME, vortexed for 30 seconds (sec), drawn ten times through a 20 – 23 gauge needle attached to a syringe, vortexed and placed on ice for 2 min. Mechanical transformation of parasites was confirmed via microscopy. To separate heads from tails, the suspension was loaded onto a 60% Percoll (Sigma-Aldrich, Saint Louis, MO, USA) in BME gradient and centrifuged for 10 min at 300 × g with minimal brake. The pellet at the bottom, containing the schistosomula, was taken up with a transfer pipette and washed three times as described above. Lastly, schistosomula were counted, resuspended in the appropriate medium and further processed.

### Schistosomula screening

Newly transformed schistosomula were resuspended in modified Basch media (Basch, 1981) (BME supplemented with 5% (v/v) heat inactivated FBS (Gibco), 2 mM L-glutamine (Gibco), 20 mM HEPES (Gibco), 0.2 mM glucose (Fisher Scientific, Pittsburgh, PA, USA), 500 nM hypoxanthine (Merck Darmstadt, Germany), 1 mM serotonin (Merck), 1 mM hydrocortisone (Merck), 200 nM triiodothyronine (Merck), 0.5× (v/v) MEM vitamins (Gibco), 10% (v/v) Schneider’s Insect Medium (Merck), 100 mg/mL lactalbumin hydrolysate (Merck) and antibiotic antimycotic solution (ABAM; 2x concentrated, Sigma-Aldrich). Parasites were dispensed into 384-well tissue culture plates at a density of 120 parasites/well and co-cultured with compounds at 37°C with 5% CO_2_ for 72 hours or 120 hours.

The compounds kifunensine and swainsonine were screened as two dilutions (50 and 10 µM) in 0.625% PBS alongside positive (10 µM auranofin (AUR; Sigma-Aldrich) and 10 µM praziquantel (PZQ; Sigma-Aldrich) in 0.625% DMSO (Sigma-Aldrich)) and negative (0.625% DMSO and 0.625% PBS) controls. At least two technical replicates were included per treatment, and each experiment was performed on two separate occasions. Following incubation, compound activity was assessed using the high-throughput, high content imaging platform Roboworm, as described previously (Paveley et al., 2012; Whatley et al., 2019). Compounds that resulted in scores equal to, or below -0.35 for both motility and -0.15 for phenotype, were considered to be hits. Z’factors for both phenotype and motility metrics were >0.35 for all experiments.

### Schistosomula culture

Newly transformed schistosomula were resuspended in long term culture medium (Pichon et al., 2026) and cultured at a concentration of ~2500 (T25 flask) or ~1500 (6 wells plate) schistosomula per mL. For culture maintenance, medium was refreshed twice a week: once a week 50% of the medium was replaced by fresh medium and once a week the schistosomula suspension was centrifuged for 3 min at 300 × g room temperature (W1) or schistosomula were settled by gravity (W2-W6), the supernatant was discarded and the schistosomula were resuspended in fresh medium. After one week of culture, human red blood cells (hRBCs; Sanquin, the Netherlands) were added at a concentration of 1% v/v. During culture maintenance, hRBCs were supplemented during each medium replacement. α-mannosidase inhibition was achieved through addition of the compounds kifunensine (21.5 μM; Cayman Chemical) or swainsonine (25 μM; Cayman Chemical). Phosphate-buffered saline (PBS; Frensius Kabi, Bad Homburg, Germany) was included as control. The compounds were added at the start of the culture and each time that the medium was refreshed, except for experiments in which the treatment was discontinued after one week (**Supplementary Figure 1**). Typically, per condition three biological replicates were obtained through three independent transformations performed on different batches of cercariae on different days.

### Schistosomula imaging

Each week, brightfield microscopy pictures were taken of schistosomula in culture with a 5 × objective (Leica N PLAN 5×/0.12 PH 0; Leica Microsystems, Wetzlar, Germany) on a Leica DMI 4000 fluorescent microscope (Leica Microsystems). Phenotype counts were performed on three brightfield images of one schistosomula culture by two independent, blinded researchers.

Live dye staining and confocal microscopy of schistosomula was performed as described earlier (Kuhlemaijer et al., 2026). In brief, CellMask Green Actin Tracking Stain (Invitrogen, Waltham, MA, USA), CellMask Plasma Membrane Stain Deep Red (Invitrogen) and NucBlue Live ReadyProbes Reagent (Invitrogen) were added to the schistosomula culture and incubated overnight. After fixation with 4% formaldehyde (Sigma-Aldrich) in PBS and three washes with PBS, schistosomula were resuspended in PBS and transferred with a micropipette to adhesive glass microscopy slides (SuperFrost, Knittel Glass, Braunschweig, Germany). Once the liquid evaporated, the schistosomula were coated with prolonged glass antifade mountant (Invitrogen) and covered with a glass cover slip (Menzel-Gläser, Braunschweig, Germany). Finally, schistosomula were visualised with a 63 × objective (Leica HC PL APO CS2 63×/1.40na OIL) by a Leica true confocal scanning (TCS) SP8 white light laser (WLL) microscope (Leica Microsystems, Wetzlar, Germany).

### N-glycan analysis

Approximately 4,000 schistosomula were harvested, washed three times in PBS and homogenised in PBS with three 2.38 mm metal beads (Qiagen, Hilden, Germany) in a bead beater (TissueLyser LT, Qiagen) at 50 Hz for 5 min, and subsequently sonicated in an ultrasonic bath (45 kHz; 146 VWR, Radnor, PA, USA), lyophilised, resuspended in PBS and sonicated. Release of N-glycans by peptide-N-glycosidase F (PNG-F) was performed as described earlier (Smit et al., 2015; Kuhlemaijer et al., 2026). In short, N-glycans were released by PNG-F (Roche, Basel, Switzerland) and loaded onto a C18 cartridge (J.T. Baker, Phillipsburg, NJ, United States). Then, the C18 flowthrough was used for purification on carbon SPE cartridges (Merck), from which the eluate was dried and redissolved in Milli-Q water (MQ) and used for labelling with anthranilic acid (AA; Sigma). Subsequently, the AA-labelled N-glycans were purified by bio-gel P-10 (Bio-Rad, Hercules, CA, USA), concentrated, redissolved in MQ and spotted on a 384-well polished steel target plate (Bruker, Billarica, MA, USA). Additionally, exoglycosidase digestions were performed as described before (Kuhlemaijer et al., 2026). In brief, AA-labelled N-glycans were digested with hexosaminidase (New England Biolabs (NEB), Ipswich, MA, USA) and β1-4 galactosidase (NEB) overnight at 37 °C. Treated glycans were spotted on the target plate by C18 ziptip (ZipTip 0.2 µL C18 resin, Merck) as described before (Kuhlemaijer et al., 2026).

### MALDI-TOF MS and mass spectra analysis

Analysis of AA-labelled N-glycans was performed as described previously (Kuhlemaijer et al., 2026). Briefly, matrix-assisted laser desorption/ionisation time-of-flight (MALDI TOF) mass spectrometry (MS) was performed on a rapifleX mass spectrometer (Bruker) with flexControl software (version 4.0; Bruker). Next, mass spectra were annotated with flexAnalysis software (version 4.0; Bruker), while peaks with a signal-to-noise (S/N) ratio below 3 were discarded. Mass-to-charge (m/z) ratios of annotated peaks were translated to possible glycan structures with the GlycoPeakfinder tool of GlycoWorkBench (Ceroni et al., 2008) and selected based on literature (Smit et al., 2015) and exoglycosidase digestions.

The obtained mass spectra contained host-derived N-glycans as well, originating from the human serum and hRBCs in the culture medium. To correct for these N-glycans, PNG-F N-glycan release and AA-labelling was performed on culture medium containing hRBCs (n = 3). Each sample was spotted in triplicate on the target plate by C18 ziptip and measured and annotated as described above. First, of each N-glycan found in a culture medium-derived mass spectrum, a correction factor was calculated, based on the sum S/N of the three most abundant sialylated marker structures: H5N4S1-AA (m/z = 2051), H5N4S1F1-AA (m/z = 2197) and H5N5S1F1-AA (m/z = 2400). The correction factor was calculated as the S/N of a found N-glycan divided by the sum S/N of the three marker structures, based on the in the culture medium-derived mass spectra. Next, for each parasite-based mass spectrum, the sum S/N of the three marker structures was determined. For each N-glycan, the correction factor was multiplied by the sum S/N to find the host contribution. This host contribution was subtracted from the total S/N of that N-glycan, to establish the parasite contribution. Secondly, a correction was done for parasite N-glycans of which the monoisotopic (M) peak overlayed with the second (M + 1) peak of a sialylated N-glycan, such as H5N4S1-AA (m/z = 2051) and H5N4F2-AA (m/z = 2052). To do this, the same medium mass spectra were used as for the first correction. Of each overlapping peak set, a correction factor was calculated by dividing the second peak’s S/N by the first peak’s S/N based on the culture medium mass spectra. In the parasite mass spectra, the correction factor of a overlapping peak set was multiplied by the monoisotopic peak’s S/N to identify the host contribution. This was then subtracted from the second peak’s S/N to obtain the parasite’s contribution. To complete the correction, all corrected N-glycans were inspected and negative values for S/N were removed. Additionally, sialylated N-glycans were removed from the list of identified glycans. Subsequently, the relative presence in percentages was calculated for each parasite N-glycan, i.e. the individual S/N was divided by the sum S/N of all identified parasite N-glycans. Finally, a quality control was performed; only N-glycans that were in at least one condition present in at least two out of three repeats and with a sum of the three repeats of ≥ 1%, were included in the final analysis. The final selection was used for graphical representation. Here, N-glycans were categorised as paucimannose, oligomannose, hybrid, hybrid and/or complex, complex or GlcNAc1 free N-glycans (FNGs) (**Supplementary Figure 2**). The category hybrid and/or complex comprised N-glycans of which, based on MALDI-TOF MS data, it was uncertain whether they were hybrid or complex. For W0-W2 PBS schistosomula, these structures were categorised as complex based on literature (Smit et al., 2015). For other samples, exoglycosidase digestions with hexosaminidase and β1-4 galactosidase were used to determine whether the hybrid and/or complex category consisted of only hybrid, only complex or a mixture of structures. This identification was based on the representative N-glycan H5N4F1-AA (m/z = 1906), since this glycan had the highest presence of all glycans in the category hybrid and/or complex.

Finally, the abundance of each glycan was calculated by normalising the (corrected) S/N ratio of the identified glycan to the total S/N ratio of all identified glycans per sample, providing a percentage (%) of all N-glycans. These percentages were used to generate graphs in GraphPad (Boston, MA, USA).

### SDS page and Western and lectin blot

Approximately 3,000 to 10,000 schistosomula were homogenised in PBS by bead beating for five times 1 min at 50 Hz and kept on ice for 2 min in between. Next, samples were sonicated for 20 min in ice cold water and protein concentrations were measured by spectrophotometer (EzDrop1000, Blue-Ray Biotech, New Tapei City, Taiwan). An equivalent of 20 µg protein was dissolved in Laemmli sample buffer with 6% (w/v) DTT, vortexed and incubated at 95 °C for 10 min and used for protein separation by SDS Page followed by Western or lectin blotting on a polyvinylidene fluoride (PVDF) membrane (GE Healthcare, Chicago, IL, USA) as described before (Kuhlemaijer et al., 2026). In addition, colloidal blue (Invitrogen) staining was performed on a separate SDS PAGE gel. For recognition of specific glycan motifs, the membranes were first blocked with tris-buffered saline supplemented with 0.1% (v/v) Tween20 and 0.5% (w/v) bovine serum albumin (TBSTB). Next, the membranes were incubated with the in-house produced monoclonal antibody 100-4G11 which binds trimannosyl groups on N-glycans (van Dam et al., 1993; van Remoortere et al., 2003) or Concanavalin A (Con A, 1:100,000; Vector Laboratories, Newark, CA, USA), followed by an anti-mouse polyvalent immunoglobulins (G,A,M)−alkaline phosphatase antibody (1:1,000, produced in goat; Sigma-Aldrich) or streptavidin-alkaline phosphatase conjugate (1:1,000; Roche). Antibody and lectin binding was visualised through incubation with 5-Bromo-4-chloro-3-indolyl-phosphate (BCIP; Roche) and 4-nitro blue tetrazolium chloride (NBT; Sigma-Aldrich).

## Results

### No immediate toxicity effects of α-mannosidase inhibitors on freshly transformed schistosomula

To test whether kifunensine and swainsonine are suitable for N-glycosylation manipulation during long term culture and to rule out direct effects from these inhibitors on the parasites, we first assessed whether the α-mannosidase inhibitors induce immediate toxicity in newly transformed schistosomula. Directly after transformation, schistosomula were cultured for three or five days in the presence of the compounds kifunensine or swainsonine, or PBS as a control. The parasites were computationally scored for motility and for phenotype, after 72 (**Supplementary Figure 3**) or 120 hours (**Figure 1**). For all experiments, the motility and phenotype of the kifunensine- and swainsonine-treated schistosomula were unaffected. Their scores clustered together with the negative controls (DMSO- and PBS-control schistosomula), while the positive controls (auranofin- and praziquantel-treated schistosomula) scored aberrantly for both motility and phenotype. Thus, the α-mannosidase inhibitors did not result in diminished vitality during five days of *in vitro* exposure.

**Figure 1:**
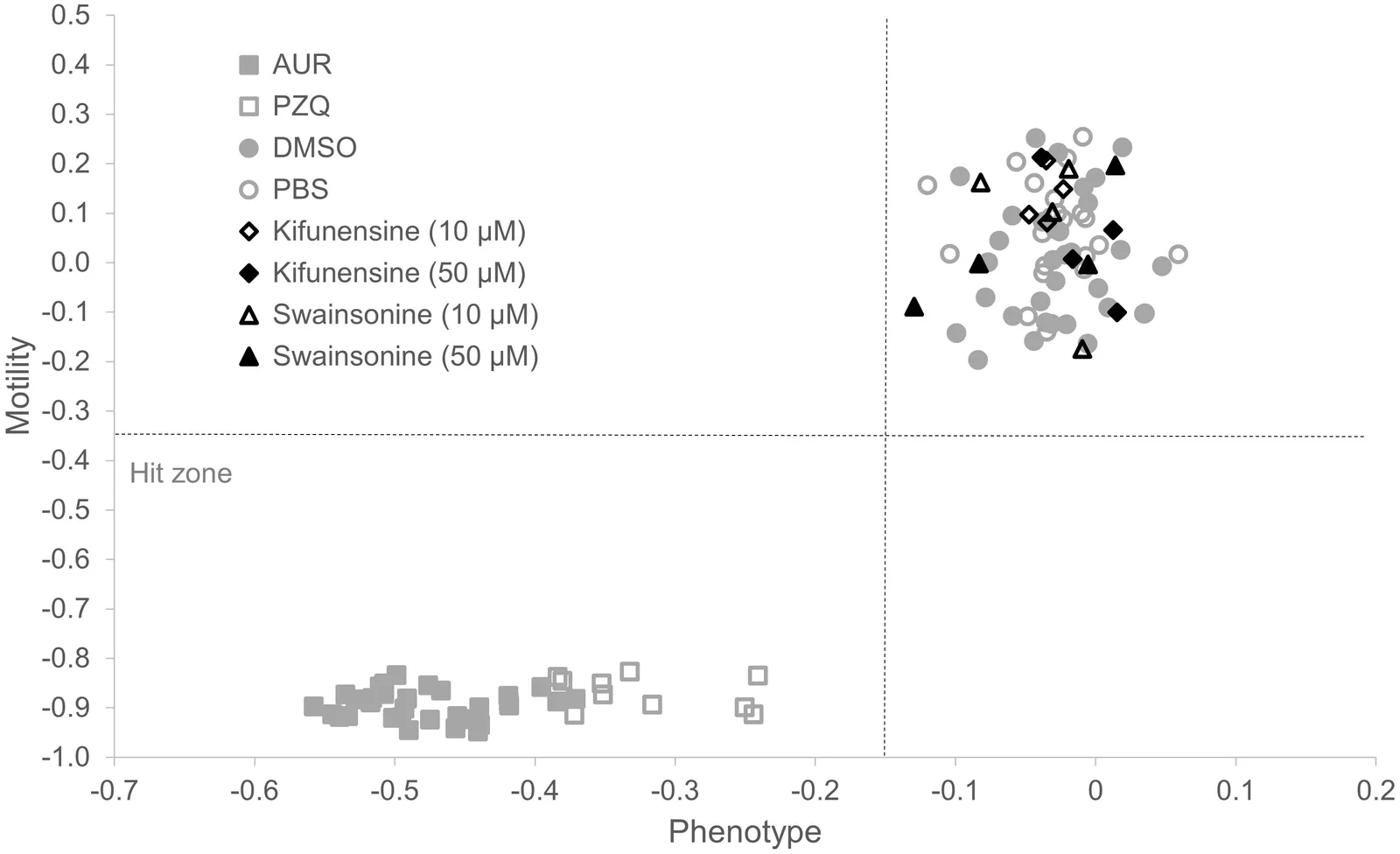
Motility and phenotype scores of PBS-control and α-mannosidase inhibitor-treated schistosomula after 120 hours. Freshly transformed schistosomula were incubated with the α-mannosidase inhibitors kifunensine or swainsonine, PBS-control or DMSO as negative controls and auranofin (AUR, 10 µM) or praziquantel (PZQ, 10 µM) as positive controls. The kifunensine- and swainsonine-treated as well as the PBS-control and DMSO parasites were not affected in motility or phenotype after 120 hours. Each point represents the mean scores of one well containing 100-120 individuals. Data from two separate experiments, each containing two technical replicates are shown. Z’ factors for both motility and phenotype metrics for these experiments were >0.48.

### N-glycan profile changes through α-mannosidase inhibition during long term culture of schistosomula

After having established that exposure of schistosomula to α-mannosidase inhibitors did not lead to immediate effects on viability, we continued to perform N-glycan remodelling though α-mannosidase inhibition during long-term culture. We examined the effect of continued α-mannosidase inhibition on the N-glycan profile during a period of six weeks, in which the schistosomula gradually mature. Weekly samples were taken from the PBS-control and treatment groups to analyse their N-glycosylation profile by MALDI-TOF MS (**Supplementary Figure 1**).

PBS-control schistosomula showed a quick shift in N-glycan types during the first week of culture (**Figure 2A**). While newly transformed schistosomula contained a median of 84.9% complex, 10.5% oligomannose and 4.6% paucimannose N-glycans, the PBS-control week 1 (W1) parasites had less complex (59.8%) and more oligomannose (30.6%), paucimannose (6.9%) and hybrid (2.7%) N-glycans. After the first week, the N-glycan types stabilised, with 41.7% complex, 43.3% oligomannose, 10.2% paucimannose and 3.9% hybrid glycans in W6 PBS-control schistosomula. Exo-glycosidase digestions showed that in PBS-control schistosomula, the N-glycan compositions that could theoretically represent both hybrid and complex structures, were in fact all representing complex structures, as shown for W3 – W6 (**Supplementary Figures 4A–D**). Kifunensine treatment resulted in an increase of oligomannose N-glycans within the first week when compared to PBS-control parasites, resulting in 72.2% at W1. Complex N-glycans had reduced to 21.3%, while paucimannose N-glycans made up 5.2% of all N-glycans. During the following five weeks, oligomannose N-glycans increased to 93.0% at W6. In W1 swainsonine-treated schistosomula, GlcNAc1 FNGs (16.4%) first appeared and hybrid (11.4%) and hybrid and/or complex (10.3%) N-glycans built up in comparison to the other conditions. Furthermore, 44.4% of all N-glycans was oligomannose, 12.2% paucimannose and 5.3% complex. Enzymatic digestions of W3 swainsonine indicated that the N-glycans of the category hybrid and/or complex N-glycans were indeed a mixture of both (**Supplementary Figure 4E**). Based on this, we reasoned that in W1 and W2 swainsonine-treated schistosomula, these N-glycans were also a mixture of hybrid and complex N-glycans. In W4 – W6 swainsonine-cultured schistosomula, however, the hybrid and/or complex N-glycans had evolved to all hybrid (**Supplementary Figures 4F–H**). At W6, the spectrum showed 25.4% GlcNAc1 FNGs, 13.3% hybrid and 5.6% hybrid and/or complex structures, supplemented by 49.5% oligomannose, 5.7% paucimannose and 0.4% complex N-glycans.

**Figure 2:**
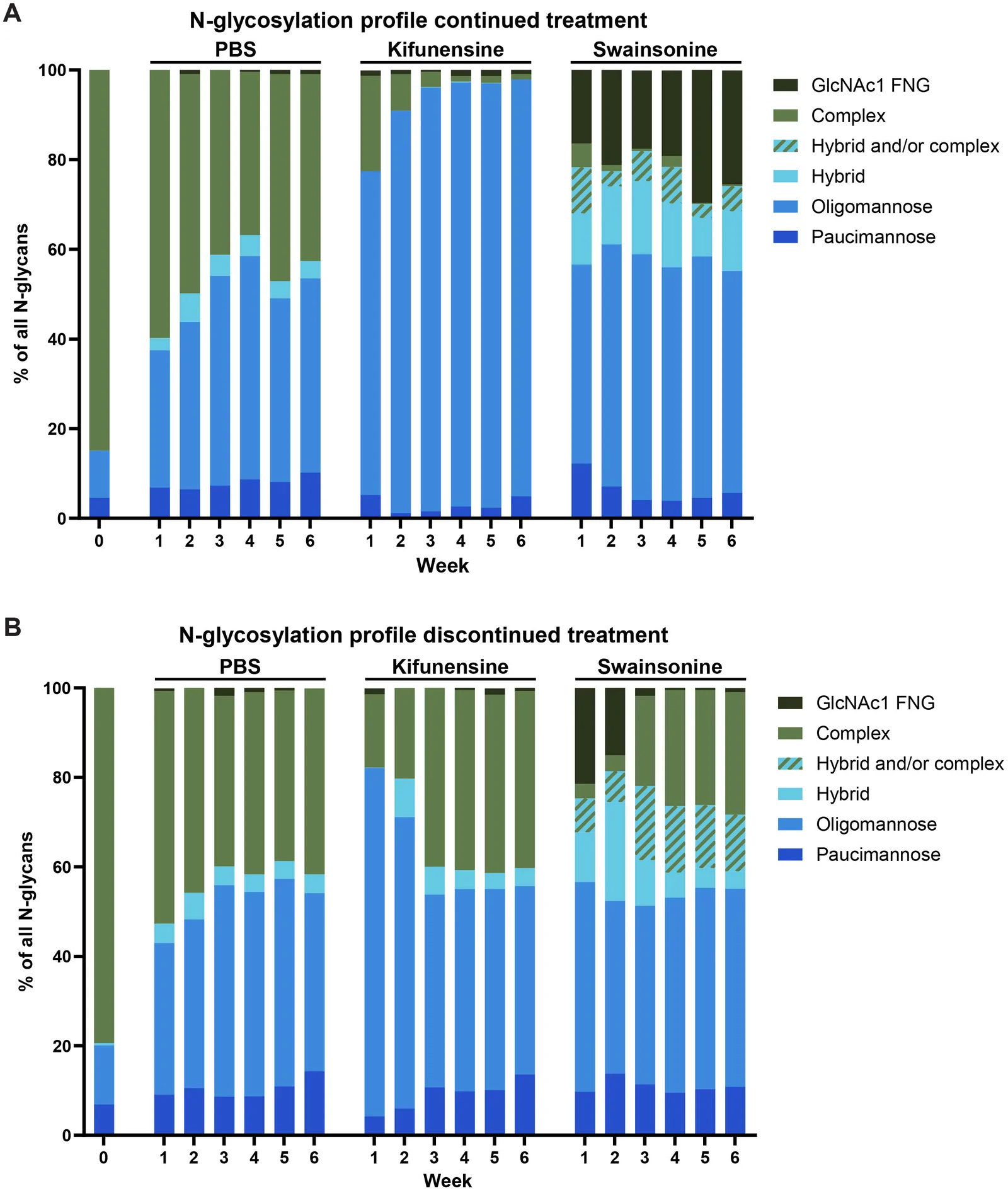
N-glycosylation profiles of freshly transformed (W0) as well as PBS-control, kifunensine- and swainsonine-treated *in vitro* cultured schistosomula. N-glycan profiles divided into paucimannose, oligomannose, hybrid, hybrid and/or complex, complex N-glycans and GlcNAc1 free N-glycans (FNG) are depicted for freshly transformed (W0) as well as W1 to W6 of continued PBS-control, kifunensine- and swainsonine-treated schistosomula **(A)** and schistosomula for which the treatment was discontinued after W1 **(B)**. The category hybrid and/or complex contains N-glycans of which it is uncertain what the configuration is, as explained in the main text. The median of three biological replicates is shown.

Next, we tested whether the N-glycan profile would recover following discontinuation of the treatment after W1 (**Supplementary Figure 1**). The profiles of the discontinued kifunensine- and swainsonine-treated parasites returned to a profile similar to that of PBS-control schistosomula. At W2, hybrid N-glycans (8.6%) appeared in the discontinued kifunensine treatment and the percentages of oligomannose N-glycans had decreased to 65.1%. One week later, the levels of all the different N-glycan categories were comparable to those of schistosomula in the PBS-control groups. Discontinued swainsonine-treated schistosomula showed an initial further increase of hybrid N-glycans to 22.1% in W2, compared to 11.1% in W1. At the same time, GlcNAc1 FNGs decreased to 15.1% in W2 versus 21.5% in W1. At later time points, the GlcNAc1 FNGs declined to the same levels as in the other conditions. The paucimannose, oligomannose and hybrid N-glycans expression levels became similar to PBS-control and discontinued kifunensine-treated schistosomula. The last category, hybrid and/or complex N-glycans, consisted of a mixture of hybrid and N-glycans at W3 and W4 (**Supplementary Figures 4I, J**), while at W5 and W6 these N-glycans were all complex (**Supplementary Figures 4K, L**).

Developing schistosomula differentially express several N-glycan motifs, such as the core modifications β1-2xylose, α1-6fucose and the antenna elements LacNAc (LN; Galβ1–4GlcNAc) and LacdiNAc (LDN; GalNAcβ1-4GlcNAc) (Smit et al., 2015). These motifs may play a role in parasite development or parasite-host interaction, for example as antibody target (Nyame et al., 2003; van Diepen et al., 2012) or ligand of lectins (van den Berg et al., 2004). Therefore, we used the MALDI-TOF MS data to analyse the presence of these specific developmentally regulated N-glycan motifs (**Figure 3**). Newly transformed schistosomula were marked by a high abundance of N-glycans with core-xylose (58.4%) and core-fucose (87.1%) (**Figure 3A**). Additionally, 41.0% of N-glycans had an LN motif, while the LDN motif was present on only 2.4% of all N-glycans (**Figure 3B**). During culture, the presence of N-glycans containing core-xylose in the PBS-control group decreased to around 1% or less from W3 onwards. Meanwhile, core-fucosylation initially reduced during culture to 44.6% in W4, after which it stabilised. N-glycans containing LN declined during culture as well, with 14.4% of all N-glycans having LN at W6. At the same time, more N-glycans with the LDN motif were detected over time, with 8.4% at W1 and 31.0% at W6.

**Figure 3:**
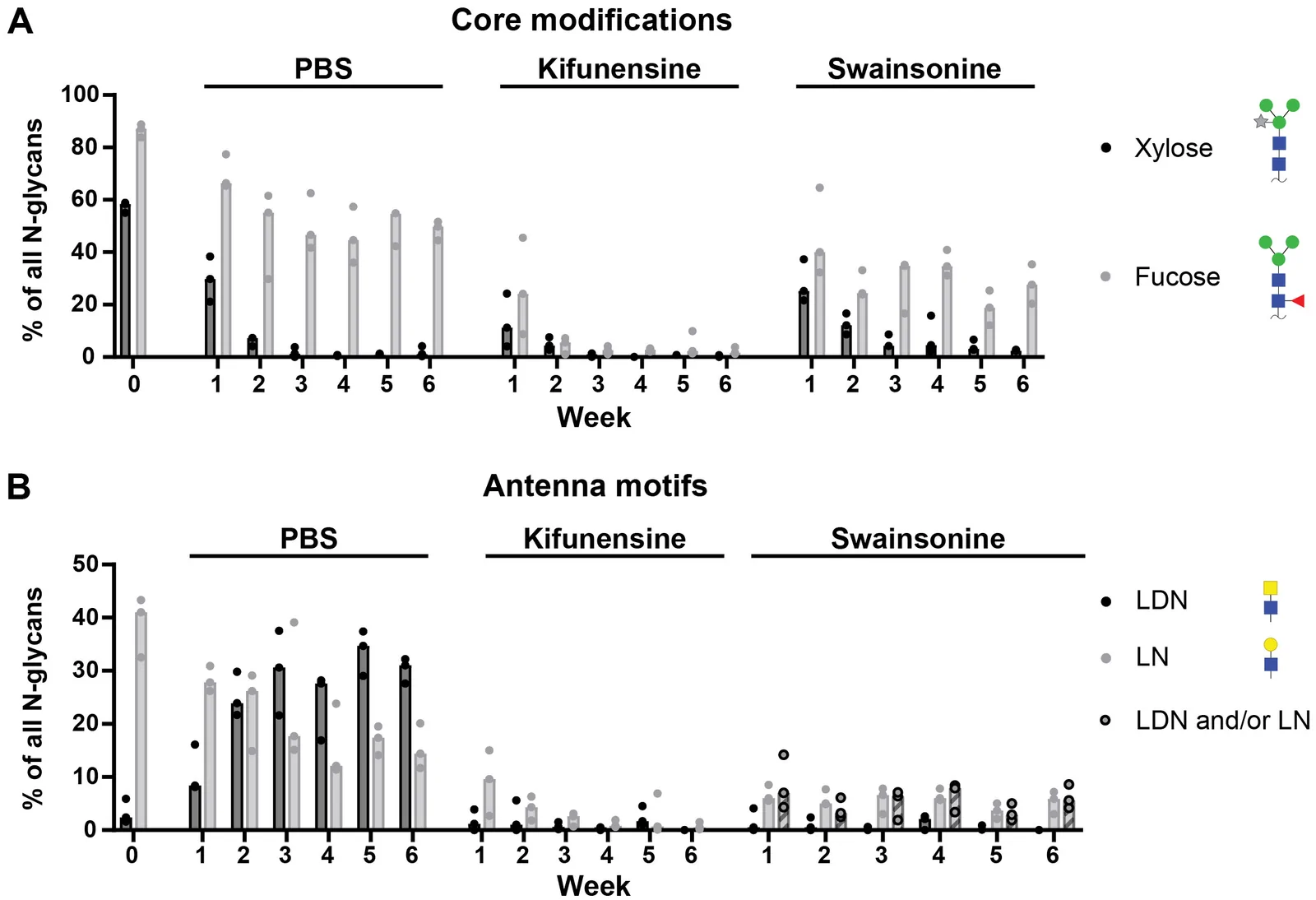
Presence of N-glycan motifs of freshly transformed (W0) as well as PBS-control, kifunensine- and swainsonine-treated schistosomula. Presence of the N-glycan motifs core-xylose and core-fucose **(A)**, and LacdiNAc (LDN; GalNAcβ1-4GlcNAc) and LacNAc (LN; Galβ1–4GlcNAc) **(B)**, are shown for W0 schistosomula and W1 to W6 of PBS-control, kifunensine- and swainsonine-treated schistosomula. The median of three biological replicates is shown with dots representing individual biological replicates. Green circle = mannose, yellow circle = galactose, yellow square = N-acetylgalactosamine, blue square = N-acetylglucosamine, red triangle = fucose, grey star = xylose.

Treatment with the α-mannosidase inhibitors decreased the presence of N-glycans with core-modifications as well as with LN and LDN motifs. In kifunensine-treated schistosomula, core-xylose was present in 11.2% of all N-glycans at W1 which developed into 1% or less from W3 onwards, and core-fucosylation dropped from 24.1% at W1 to around 3% or less at W3 and later time points (**Figure 3A**). Both reductions were due to the decrease of core-modified complex N-glycans (**Supplementary Figure 5**). In line with the decreasing complex N-glycans in kifunensine-treated parasites, the LN motif disappeared from 9.6% at W1 decreasing to 0.5% at W6, and the development of the LDN motif was prevented (**Figure 3B**). The total presence of core-modifications was less affected by swainsonine than by kifunensine (**Figure 3A**). Core-xylose presence over time showed similarities with PBS-control schistosomula, as it diminished gradually, and core-fucose levels were approximately 20% lower than in PBS-controls. However, core-modifications were detected on hybrid, hybrid and/or complex and oligomannose N-glycans in swainsonine-treated parasites, instead of paucimannose and complex N-glycans in PBS-control schistosomula. LN and LDN presence was clearly affected by swainsonine treatment (**Figure 3B**), as these are mostly found on complex N-glycans. The LN motif was continuously present at low levels (between 3.7% and 6.6%) and LDN was mostly absent. N-glycans with an LDN and/or LN motif, which is uncertain because they belong to the hybrid and/or complex category, had a relatively stable median presence between 2.9% and 7.9%.

Discontinuation of treatment after W1 caused both the kifunensine- and swainsonine-treated schistosomula to rapidly return towards a core-modification profile comparable to PBS-control parasites from W3 onwards (**Supplementary Figure 6A**). This trend was also seen for the LDN and LN motifs; LDN levels increased in both discontinued kifunensine- as well as swainsonine-treated parasites from W2 onwards. The presence of LN initially grew until W3, after which LN percentages stabilised (**Supplementary Figure 6B**).

Additionally, we examined the presence of specific N-glycan markers for kifunensine and swainsonine treatment. We based the selection of markers on our previous study in adult worms (Kuhlemaijer et al., 2026): oligomannose N-glycans with nine (M9) or eight (M8) mannoses as markers for kifunensine (**Figure 4A**) and oligomannoses with five mannoses (M5), including core-fucosylated (M5F1), core-xylosylated (M5X1) and M5X1F1, as markers for swainsonine (**Figure 4B**). W0 schistosomula had a median 3.7% M9 and 1.5% M8 N-glycans. Both increased in PBS-control as well as swainsonine-treated parasites, resulting in 14.8% M9 and 8.2% M8 in PBS-control and 9.0% M9 and 4.7% M8 in swainsonine treatment at W6. In kifunensine-treated schistosomula, M9 made up 42.8% of the N-glycosylation profile at W1 and stayed high until W6 (47.4%). M8 formed 14.5% of all W1 N-glycans in the kifunensine treatment and followed a slightly increasing trend towards 24.0% at W6. (Core-modified) M5 was virtually absent (0.8%) at W0, increased slightly in PBS-control and kifunensine treatment to 2.4% and 6.4% at W6, respectively. Only M5 and M5F1 were found in these two conditions, but no M5X1 and M5X1F1 were observed. Meanwhile, swainsonine-treated parasites contained more (core-modified) M5 at W1: 12.4% M5, 5.1% M5F1, 4.0% M5X1 and 2.5% M5X1F1. This developed into 15.5% M5, 10.1% M5F1, 1.4% M5X1 and 0.3% M5X1F1 at W6.

**Figure 4:**
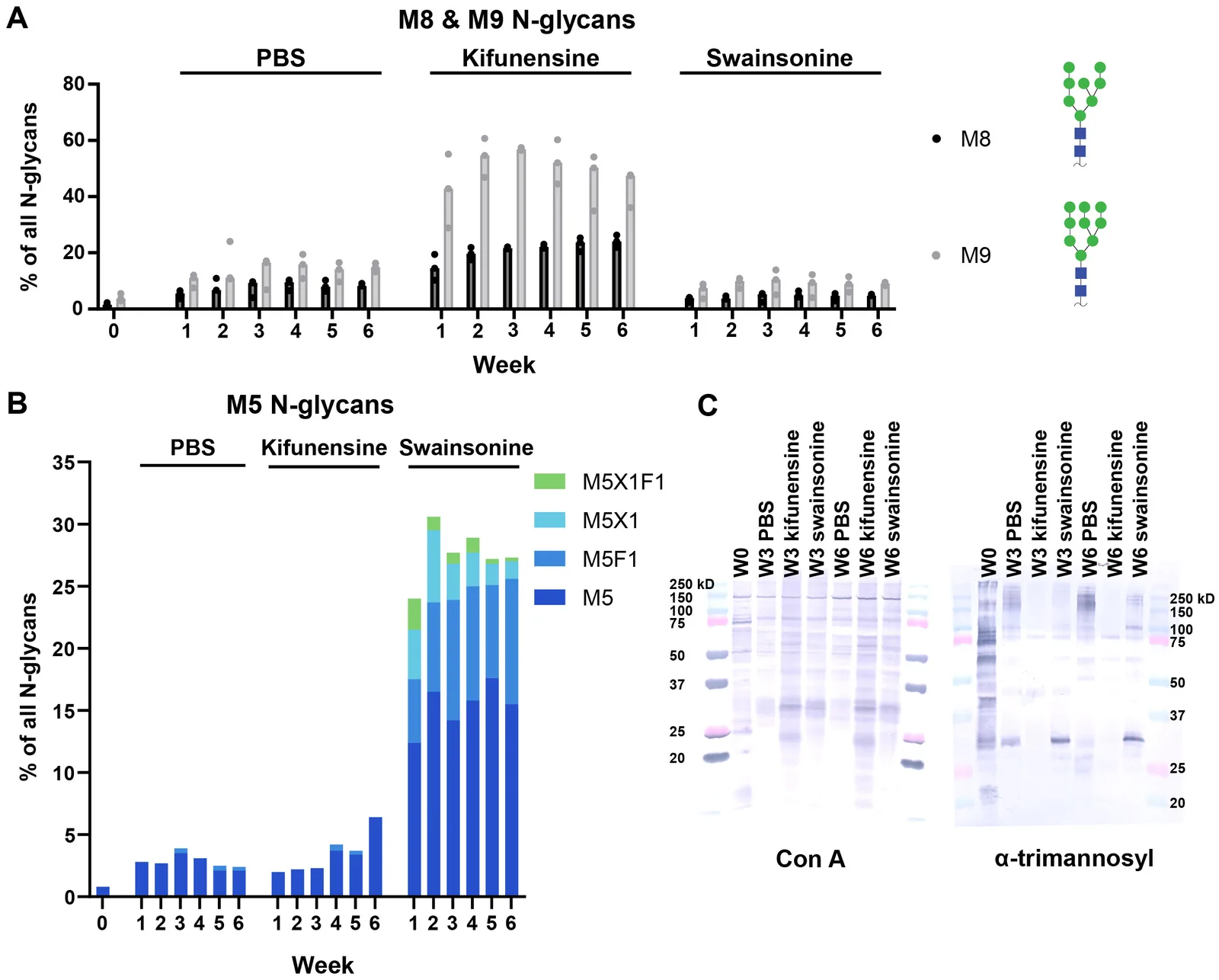
Presence of α-mannosidase inhibition-specific markers in freshly transformed (W0) as well as PBS-control, kifunensine- and swainsonine-treated schistosomula. The kifunensine-specific markers M9 (Man9GlcNAc2) and M8 (Man8GlcNAc2) **(A)**, and swainsonine-specific marker (core-modified) M5 (Man5GlcNAc2) **(B)** are shown for W0 schistosomula and W1 to W6 of PBS-control, kifunensine- and swainsonine-treated schistosomula. The median of three biological replicates is shown with dots representing individual biological replicates **(A, B)**. Lectin and Western blots containing protein extracts of W0, W3 and W6 schistosomula were incubated with con A, a lectin with affinity for mostly high mannose (M7-M9) N-glycans, and an antibody for trimannosyl (Manα1-3(Manα1-6)Man). A representative blot of two biological replicates is shown **(C)**. Green circle = mannose, blue square = N-acetylglucosamine.

To investigate the changes in mannosylated N-glycans observed by MALDI-TOF MS at the glycoprotein level, we applied SDS-PAGE in combination with Western or lectin blotting (**Figure 4C**). The lectin Con A, most strongly recognising high mannose (M7-M9) N-glycans (Hori et al., 2007), bound more intensely to kifunensine-treated W3 and W6 parasites, showing additional glycoprotein bands when compared to PBS-control, swainsonine-treated schistosomula and W0 schistosomula. This observation is in line with the MALDI-TOF MS data. Moreover, the 100-4G11 antibody against the trimannosyl (Manα1-3(Manα1-6)Man) motif that is present in M3, M5 and hybrid N-glycans, revealed an intense glycoprotein band between 25 and 37 kD after swainsonine treatment, further confirming observations by MALDI-TOF MS. Again, the pattern of glycoprotein bands detected by the 100-4G11 antibody differed clearly between W0 and W3/W6.

After discontinued treatment, the kifunensine-specific markers M8 and M9 (**Supplementary Figure 7A**) and the swainsonine-specific (core-modified) M5 (**Supplementary Figure 7B**) decreased within two weeks to levels similar to the (dis)continuous PBS-control. This was seen in the lectin and Western blots too, where the differences in band patterns between the conditions were absent in W6 schistosomula (**Supplementary Figure 7C**).

Together, these results show that α-mannosidase inhibition created distinct N-glycosylation profiles at W1 for each condition, after which the treatment-characteristic N-glycan profiles were maintained. Specifically, PBS-control schistosomula had mostly oligomannose and complex N-glycans. The proportion of LDN motifs increased while LN motifs decreased during maturation of the schistosomula. Core-xyloses disappeared during culture and core-fucose stayed present, although at lower levels. Kifunensine-treated schistosomula were characterised by oligomannoses, specifically M9 and M8, and had barely any N-glycans with core-modifications. After swainsonine treatment, hybrid (and/or complex) N-glycans, GlcNAc1 FNGs and (core-modified) M5 oligomannoses marked the schistosomula N-glycan profile.

### Continued inhibition of α-mannosidase I during culture provoked oedema in schistosomula

Following analysis of the changes in parasite N-glycosylation, we assessed tentative effects on parasite development associated with the perturbed N-glycosylation resulting from the α-mannosidase inhibition by kifunensine or swainsonine.

Brightfield microscopy pictures of live schistosomula were taken during each week of culture. At W2, schistosomula in all conditions had grown and developed a bifurcated gut containing hemozoin (**Figure 5**), resembling early liver stage schistosomula (Pichon et al., 2026). At W4, the size of all parasites had increased and features including oral and ventral suckers became visible. Heterogeneity in size and development between parasites in the same culture was present in all treatment groups, including the PBS-control. In the kifunensine treatment at W4 and consecutive weeks, a phenotype was observed where a subset of the developing schistosomula showed signs of local oedema. The body was locally bloated and turned dark by what appeared to be an accumulation of hemozoin. Not all kifunensine-treated parasites were affected; at W4 this phenotype was observed in 8.2% of the schistosomula, increasing to 11.1% at W5 and 21.9% at W6 (**Supplementary Figure 8**). However, this phenotype appeared consistently over three biological replicates and was clearly less found in the PBS-control (3.4% at W6) and swainsonine-treated schistosomula (2.9% at W6). Kifunensine-treated parasites containing the oedema phenotype were still motile and contained hemozoin in their gut. At W6, schistosomula of all conditions had further increased in size and obtained a worm-like appearance. Additionally, differences in female and male schistosomula started to appear, with female parasites being longer and male being shorter with a broader head, being features of dimorphic schistosomula (Pichon et al., 2026). However, at W6 the morphological differences were not pronounced enough to systematically identify individual schistosomula as female or male, as most W6 schistosomula are still at the liver stage (Pichon et al., 2026).

**Figure 5:**
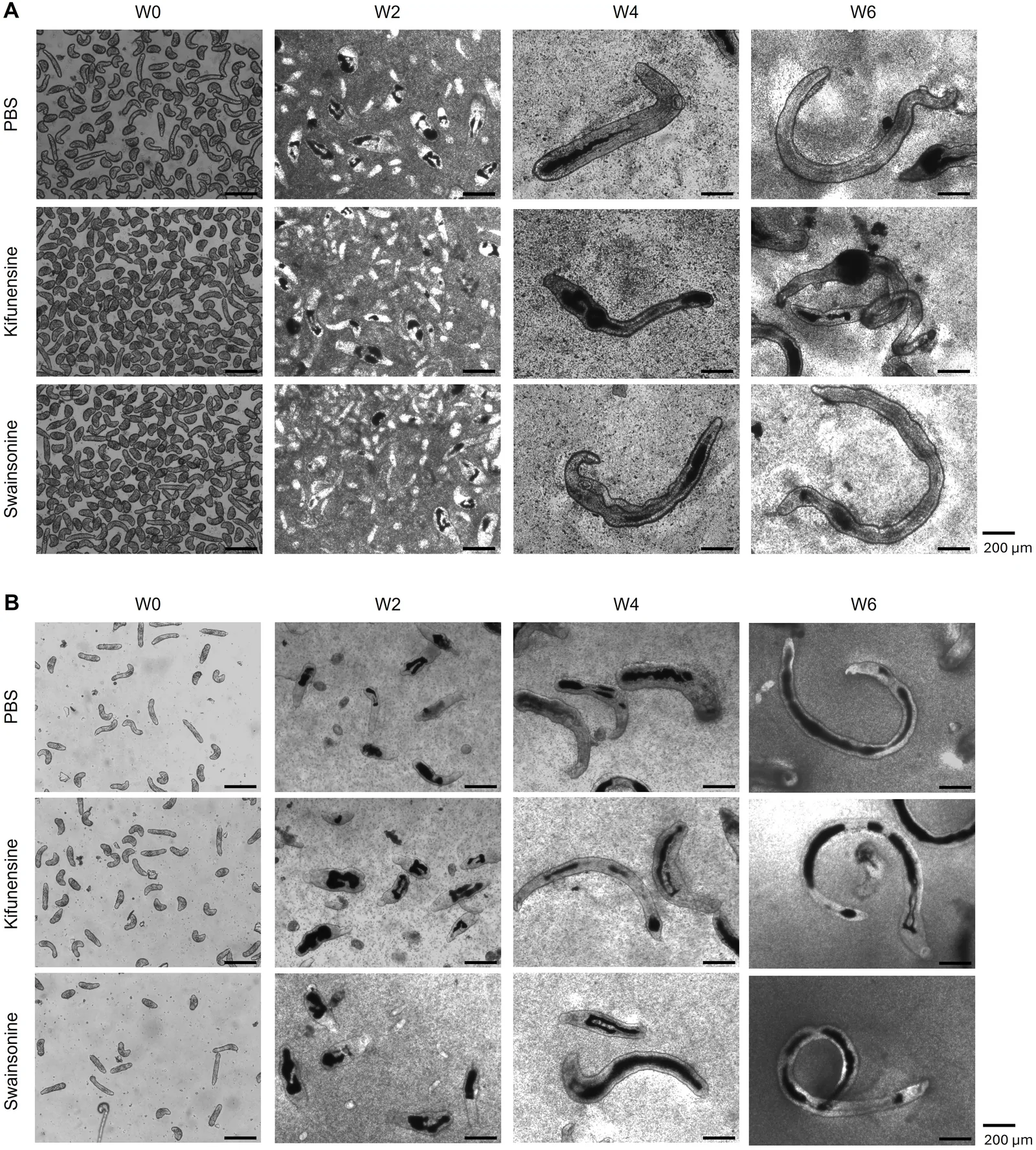
Brightfield pictures of live schistosomula. Brightfield pictures were taken every two weeks (W0, W2, W4 and W6) from schistosomula with continuous **(A)** and discontinued **(B)** treatment. A representative picture of three biological replicates is shown.

To further characterise this phenomenon, we stained the schistosomula with live dyes for nuclei, cell membranes and actin, fixed them and imaged them using confocal microscopy (**Figure 6**). Features of normal development including the oral sucker, ventral sucker, oesophagus, muscle layer and tegument were evident. At W4, local oedema became visible in kifunensine-treated schistosomula as darkened interruptions of the gut. Additionally, alterations in the swainsonine-treated parasites became visible, while they were not observed through brightfield microscopy. At W6, all previously described features of normal development were found in parasites of each condition (Pichon et al., 2026). The local oedema in the kifunensine- and swainsonine-treated parasites persisted as well.

**Figure 6:**
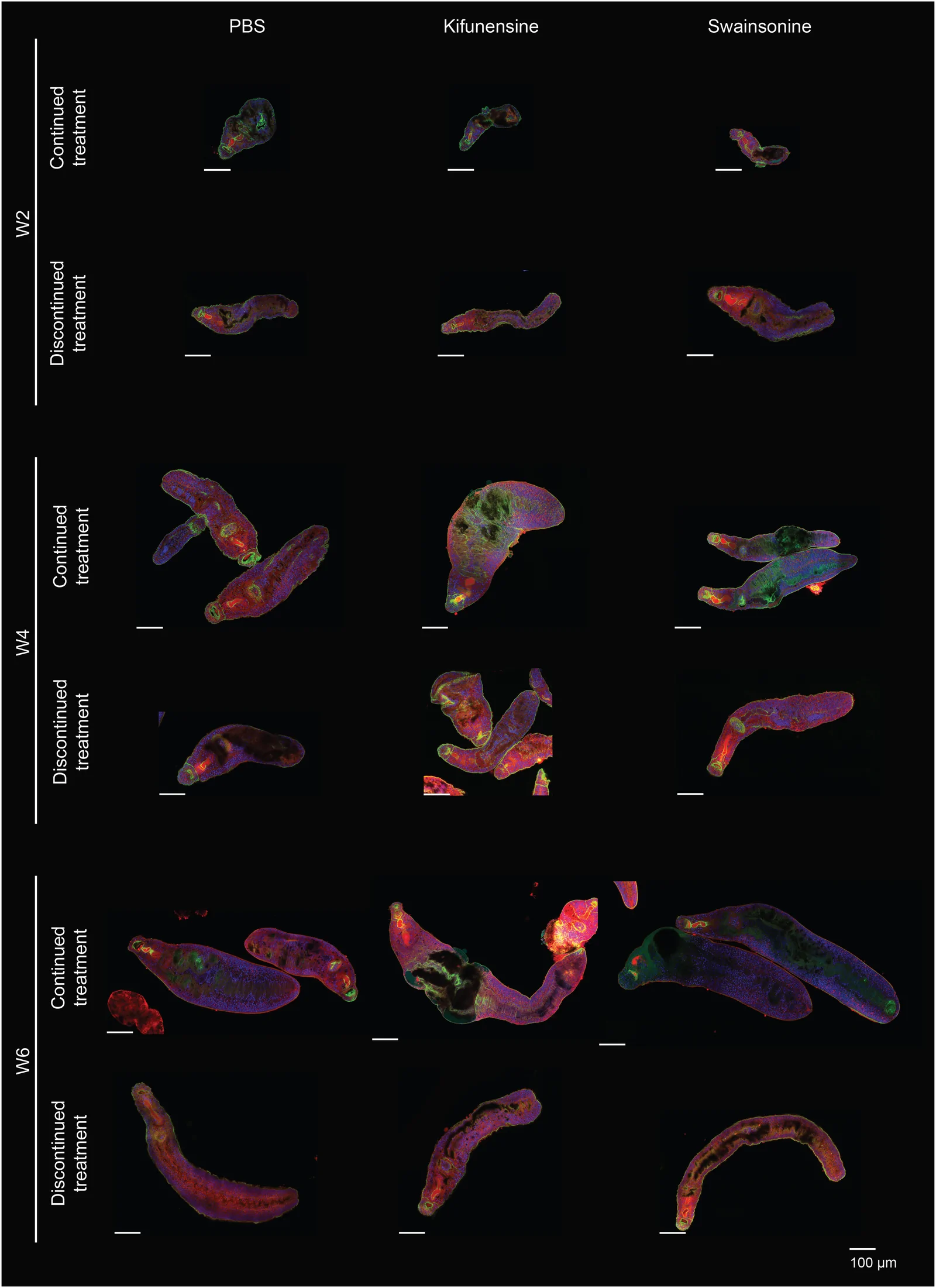
Confocal microscopy pictures of fixed live-dyed schistosomula. chistosomula were live-stained for nuclei (blue), actin (green) and membranes (red) and fixed. Confocal microscopy pictures were taken every two weeks (W2, W4, W6) of schistosomula with continued and discontinued treatment. Representative pictures of three biological replicates are shown.

In contrast to schistosomula continuously treated with α-mannosidase inhibitors, local oedema was not observed in the parasites with discontinued treatment, based on brightfield pictures (**Figure 5B**) and confocal microscopy pictures (**Figure 6**).

Taken together, α-mannosidase inhibitor-treated as well as PBS-control schistosomula underwent growth and development during the long-term culture, showing increased size and developed suckers, oesophagus, muscle layer and tegument and sexual dimorphism from W4 onwards. Additionally, a subset of the kifunensine-treated schistosomula developed local oedema, first seen at W4, but the phenotype did not appear after discontinuation of treatment after W1.

## Discussion

N-glycosylation is a fundamental process in eukaryotes and plays a role in immune modulatory processes in schistosome infections, making N-glycans a tentative target to interfere with parasite development and survival in the host. Schistosomula growth and development inside the definitive host is critical for the establishment of chronic schistosomiasis and takes several weeks (Colley and Secor, 2014). As an initial step towards a better understanding of the role of glycosylation in schistosome biology, development and parasite-host interaction, this study aimed to modify the N-glycosylation of *in vitro* cultured schistosomula and to assess the effect on parasite N-glycan expression and parasite development. As main results, we showed that treatment with the α-mannosidase inhibitors kifunensine and swainsonine resulted in high abundance of M9 N-glycans after kifunensine treatment, and M5 and hybrid N-glycans and GlcNAc1 FNGs after swainsonine treatment. While neither inhibitor invoked immediate toxicity, schistosomula development was affected by kifunensine, leading to localised oedema that seemed associated with the gut. Interestingly, upon discontinuation of the treatment after one week, the N-glycan profile returned to normal within two weeks and no inhibitor-specific phenotype was observed. In future studies, N-glycan remodelled schistosomula would serve as a functional tool to shine light onto the role that N-glycosylation has in schistosomula growth and development and in evasion and modulation of the host’s immune response, to ultimately reveal tentative candidates for novel drug of vaccines targets.

In this study, PBS-control parasites are a model for the *in vivo* development of the schistosomula, including the maturation of the N-glycosylation profile. In accordance with previously described N-glycosylation profiles of schistosomula (Smit et al., 2015), we observed similar changes in xylosylation and in LN and LDN expression upon *in vitro* culture (**Figure 3**). In general, the presence of LDN as well as both core-modifications in our *in vitro* data was comparable to the previously published data. Specifically, in both studies LDN was found to continuously increase during schistosomula maturation, core-xylosylated structures disappeared and core-fucosylated N-glycans reduced during further development. While percentages of core-modifications at W0 and W3 were comparable between the studies, the decrease of core-modifications from W0 to W1 was more profound in the previous study. Differences in the culture medium, e.g. the use of human serum in our study, and the use of *in vitro* versus *in vivo* grown schistosomula, could possibly explain these differences. This is particularly relevant as environmental nutrients affect the composition of the glycan profile (Becerra-Arteaga and Shuler, 2007; Rudd et al., 2022). Nonetheless, the general maturation of the N-glycan profile, including the LDN motif and core-modifications, is comparable between the two studies, showing that the *in vitro* cultured parasite N-glycosylation is comparable to that of *in vivo* grown parasites.

Furthermore, this study showed α-mannosidase inhibitor-mediated changes in schistosomula N-glycosylation that were comparable to the N-glycan changes earlier observed in adult schistosomes treated with the same inhibitors (Kuhlemaijer et al., 2026). Of these, a remarkable effect of swainsonine was the production of GlcNAc1 FNGs, containing only one instead of two GlcNAc in the N-glycan core, found in both schistosomula and adult worms (Kuhlemaijer et al., 2026). This effect of swainsonine has been reported before in mammalian cells treated with swainsonine and was attributed to the inhibition of cytosolic α-mannosidase (Crispin et al., 2006; Chantret and Moore, 2008; Morikawa et al., 2022).

In our current investigation, initial experiments showed that no immediate toxicity effects were observed on schistosomula that were *in vitro* cultured with kifunensine or swainsonine for up to 120 hours (**Figure 1**; **Supplementary Figure 3**). Based on brightfield and confocal microscopy with live dyes for cell nuclei, actin and cell membranes (**Figures 5**, **6**), no effects induced by the inhibitors or the resulting pronounced N-glycosylation changes were evident within the first four weeks of culture with continued treatment. Yet, at W4, local oedema of the gut was observed under brightfield microscopy of kifunensine-treated schistosomula (**Figure 5**), which was confirmed through confocal microscopy (**Figure 6**). Additionally, confocal microscopy of swainsonine-treated schistosomula exposed disorganisation of the gut as well (**Figure 6**). These effects were only observed in continuously treated parasites; when treatment was discontinued after one week, no abnormalities of the gut were found. This observation suggests that treatment during the initial week of culture alone, may not lead to the inhibitor-related phenotype. It is unclear whether the continued treatment leads to the phenotype due to the continuity, or due to treatment at an essential moment later in the parasite’s development. A potential mechanism behind the observed phenotype could be related to cell adhesion proteins, which function is affected by N-glycans. E-cadherin is involved in epithelial cell-cell adhesion and its N-glycosylation, both its presence and glycan type, has been shown to affect the strength of its adherence function (Liwosz et al., 2006; Pinho et al., 2011). Additionally, integrins regulate adhesion between cells and the extracellular matrix, and N-glycans play an important role in their biological function (Gu and Taniguchi, 2004). Another possible explanation could be related to the digestive function of the schistosome gut. It has been suggested that N-glycosylation of intestinal epithelial cells affects the intestinal morphology and thereby the digestion of amino acids (Wang et al., 2025). This is supported by the observation that differentiation of mammalian epithelial cells *in vitro* is accompanied by N-glycan changes, i.e. a decrease in highly mannosylated structures, and upregulation of Golgi α-mannosidases (Link-Lenczowski et al., 2019). Thus, the shift in schistosome N-glycosylation caused by α-mannosidase inhibition could hypothetically lead to tissue disorganisation through altered cell adhesion or disrupt cell differentiation. Further studies are necessary to address this issue.

*In vitro* culture and development of schistosomula highly depends on the used medium, where LTC medium provided a new breakthrough in culture success (Pichon et al., 2026). Two key components are human serum, which makes up 20% of the total volume, and human red blood cells, accounting for 1-2% of the medium. For our current study, addition of these essential additives may add confounders to the N-glycosylation analysis by MALDI-TOF MS as this results in contamination of the schistosomula N-glycans with human-derived N-glycans. We found multiple sialylated N-glycans, which are not produced by the parasite (Hokke and Deelder, 2001; Smit et al., 2015), but may have been ingested by the parasite (Skelly and Wilson, 2006) or could be present on host components that stick to the parasite. Presence of sialylated N-glycans in parasite extracts that are attributable to the host has been reported before (Chen et al., 2026). In our study, we corrected for the presence of both sialylated and non-sialylated human-derived N-glycans based on the N-glycans found in culture medium control samples. The N-glycans in the culture medium are insensitive to glycan manipulation by α-mannosidases, as no active N-glycosylation machinery is present (Moras et al., 2017). Additionally, each condition received the same culture medium and was corrected in the same fashion. Consequently, we do not expect that the correction for host N-glycans affects the comparison between the experimental conditions tested in this study.

A recent study described the general adult schistosome N-glycoproteome, revealing insights in protein- and site-specific N-glycosylation patterns (Chen et al., 2026). For instance, several schistosome glycoproteins show a high glycan heterogeneity. Whether this microheterogeneity is reduced after glycan remodelling as performed in our study remains to be explored. Additionally, it was described that the gut glycoproteins had a high N-glycan complexity but low N-glycosylation site numbers when compared to other tissues. In our study, we found that treatment with α-mannosidase inhibitors was associated with malformations in the schistosomula gut (**Figures 5**, **6**). Whether this is due to changes in N-glycosylation after α-mannosidase inhibition affecting local protein expression and function, or due to other more indirect effects, remains to be further investigated. Here, blot analyses detected changes in glycosylation on intact glycoproteins (**Figure 4C**). Specifically, oligomannoses were detected on more proteins in kifunensine-treated parasites than in the other conditions and swainsonine treatment caused an intense detection of a specific protein exposing the trimannosyl motif. Together, (N-)glycan remodelling and in-depth techniques such as glycoproteomics could show how individual glycoproteins as well as distinct tissues are affected when glycosylation is manipulated in live parasites. Through this characterisation, alterations in parasite biology or viability could potentially be linked to relevant glycoproteins.

Application of glyco-remodelled schistosomula in *in vitro* studies mimicking the host’s environment or *in vivo* studies would be informative to analyse the development of the glyco-remodelled schistosomula further. Immune components such as the complement system, innate and adaptive immune cells could provide an immune challenge that could possibly make the effect of the altered N-glycosylation and observed local oedema be of influence on the parasite’s survival. However, the quick recovery of the N-glycosylation profile observed after discontinuation of treatment indicates that glycan remodelling by α-mannosidase inhibitors is not suited to perform long term co-culture *in vitro* experiments or *in vivo* experiments (**Supplementary Figures 6**, **7**). A first approach to overcome this would be RNA interference (RNAi) to knock down the expression of enzymes involved in the N-glycosylation pathway. Although it is not known whether RNAi would lead to a more sustained effect on the parasite N-glycosylation, it is species-specific and could therefore be applied in combination with host cells or inside a host (Xie et al., 2025). Genome editing by CRISPR could be a method to create a long-lasting effect, creating a parasite with a permanently changed N-glycan profile. Previous studies have shown that CRISPR can be applied to different schistosome life stages, including schistosomula, adult worms and eggs, although efficiency can differ between life stages possibly due to expression level and/or morphological differences between the life stages (Sankaranarayanan et al., 2021; Du et al., 2023). Genome editing of eggs followed by continuation of the life cycle might be an attractive yet highly challenging approach to reach a stable knock-down throughout the life stages. The feasibility of this approach would in addition depend on the egg’s viability and the parasite’s ability to develop through all life stages (Du et al., 2023), which might be affected by α-mannosidase silencing, as seen after kifunensine treatment of developing schistosomula in this study. Another approach could be introducing new genes into the parasite’s genome, leading to (over)expression of specific glycoenzymes and thereby increased presence of certain glycan motifs (Ittiprasert et al., 2024). RNAi and CRISPR would also introduce the possibility to alter specific N-glycan motifs or motifs on other glycan types (O-glycans or GSLs), which is not possible with kifunensine or swainsonine. Glycan recognition by host receptors such as C-type lectins is often specific for a certain motif, meaning that altered parasite glycosylation could lead to a different interaction with host cells. Thus, schistosomes with a sustainably altered glycosylation would allow to study their development in a host(-like) environment and perform functional studies into the host immune recognition of and response toward specific glycans. Applying glycoengineering by RNAi or CRISPR is therefore a new horizon that could be explored in order to facilitate functional studies.

In conclusion, we showed that glycan remodelling of live schistosomula by α-mannosidase inhibition through kifunensine or swainsonine is effective, leading to parasites with a distinct N-glycan profile. Additionally, we showed that glyco-remodelling by kifunensine affects the parasite’s development in the form of local oedema, while no immediate toxicity of neither of the compounds was found. The affected N-glycosylation and development could be prevented by discontinuation of the treatment after one week, resulting in full recovery to normal N-glycosylation and development. Taken together, our study showed that N-glycosylation during *in vitro* schistosomula maturation is a dynamic process, that can be tuned to create a glyco-remodelled parasite. In future studies, glycan manipulation approaches can be used for studying the contribution of glycans in parasite development and survival inside the host, exposing tentative targets for novel control strategies for schistosomiasis and other neglected tropical diseases at large.

## Data Availability

The raw data supporting the conclusions of this article will be made available by the authors, without undue reservation.
