# Supplementary material for: Chemical inhibition of α-mannosidase in S. mansoni schistosomula leads to reshaped N-glycosylation associated with developmental alterations
Source: Front Parasitol. 2026 Sep 15;5:1930872. doi: 10.3389/fpara.2026.1930872 (PMC13619887; doi:10.3389/fpara.2026.1930872)
Supplement: Supplementary file 1 [file DataSheet1.pdf]

Supplementary material

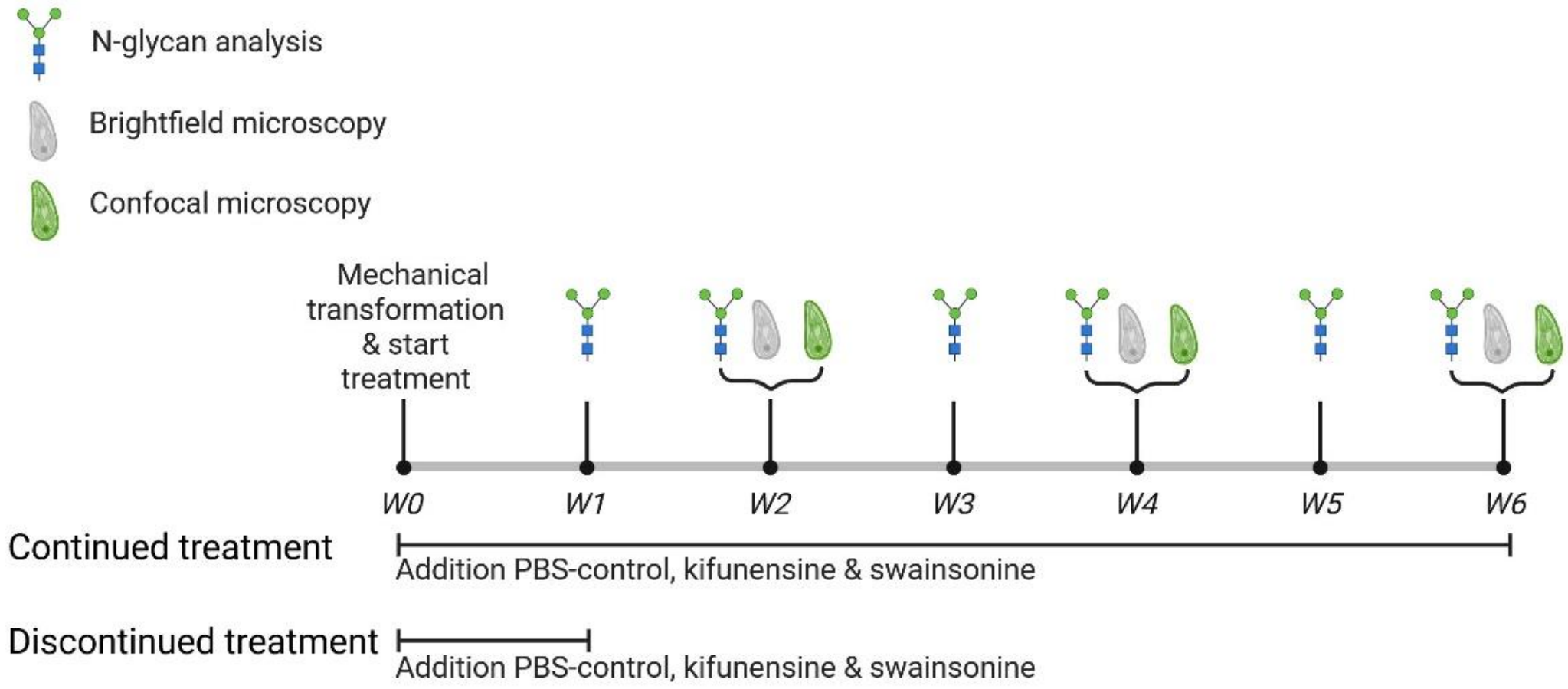

Supplementary figure 1 Overview of experimental set-up[1]

[1] Created in BioRender. Kuhlemaijer, N. (2026) https://BioRender.com/zbnqllt

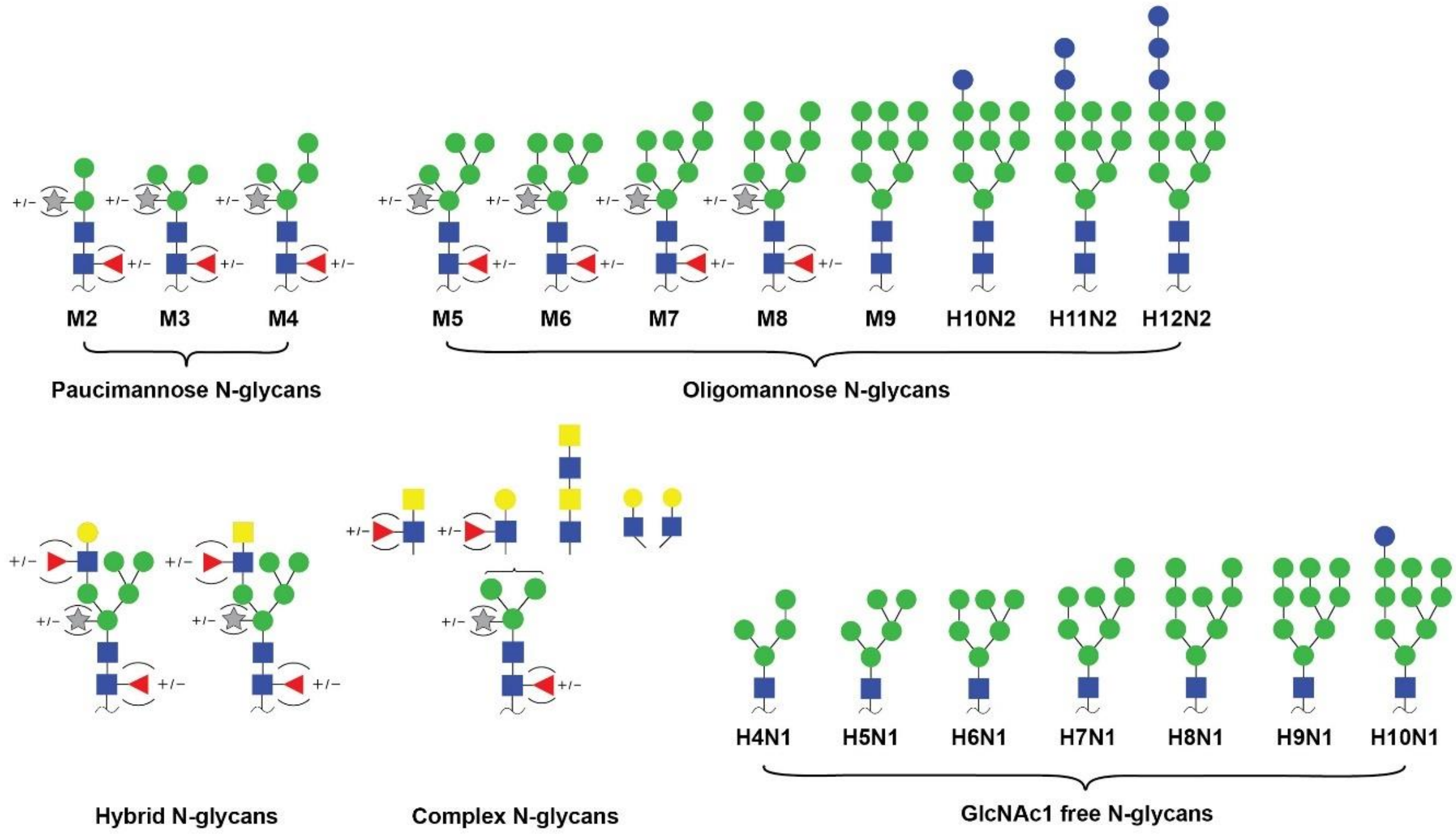

Supplementary figure 2 Overview of N-glycan categories
Green circle = mannose, yellow circle = galactose, blue circle = glucose, yellow square = N-acetylgalactosamine, blue square = N-acetylglucosamine, red triangle = fucose, grey star = xylose.

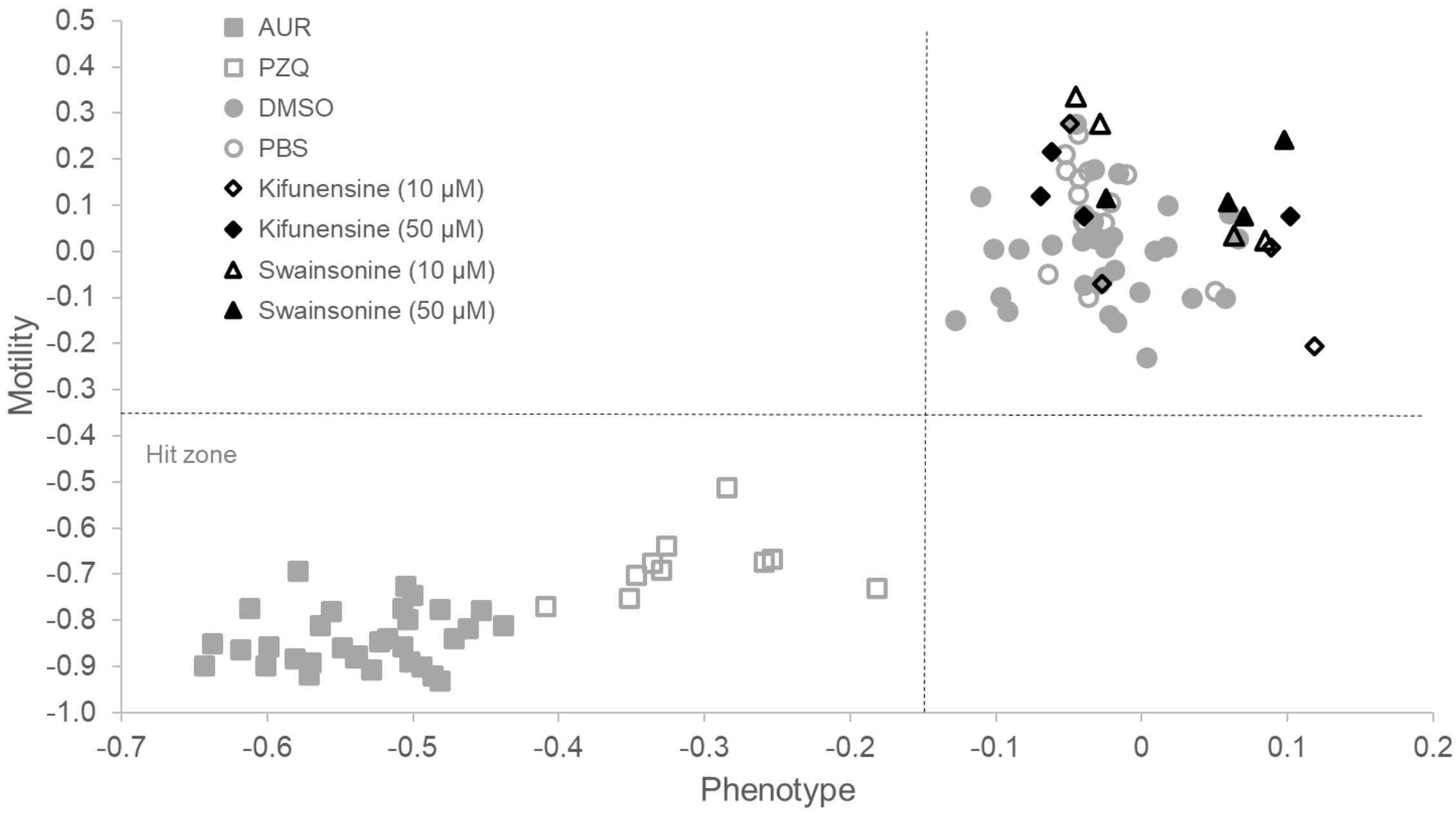

Supplementary figure 3 Motility and phenotype scores of PBS-control and α-mannosidase inhibitor-treated schistosomula after 72 hours

Freshly transformed schistosomula were incubated with the α-mannosidase inhibitors kifunensine or swainsonine, PBS-control or DMSO as negative controls and auranofin (AUR, 10 µM) or praziquantel (PZQ, 10 µM) as positive controls. The kifunensine- and swainsonine-treated as well as the PBS-control and DMSO parasites were not affected in motility or phenotype after 72 hours. Each point represents the mean scores of one well containing 100-120 individual parasites. Data from two separate experiments, each containing two technical replicates are shown. Z' factors for both motility and phenotype metrics for these experiments >0.35.

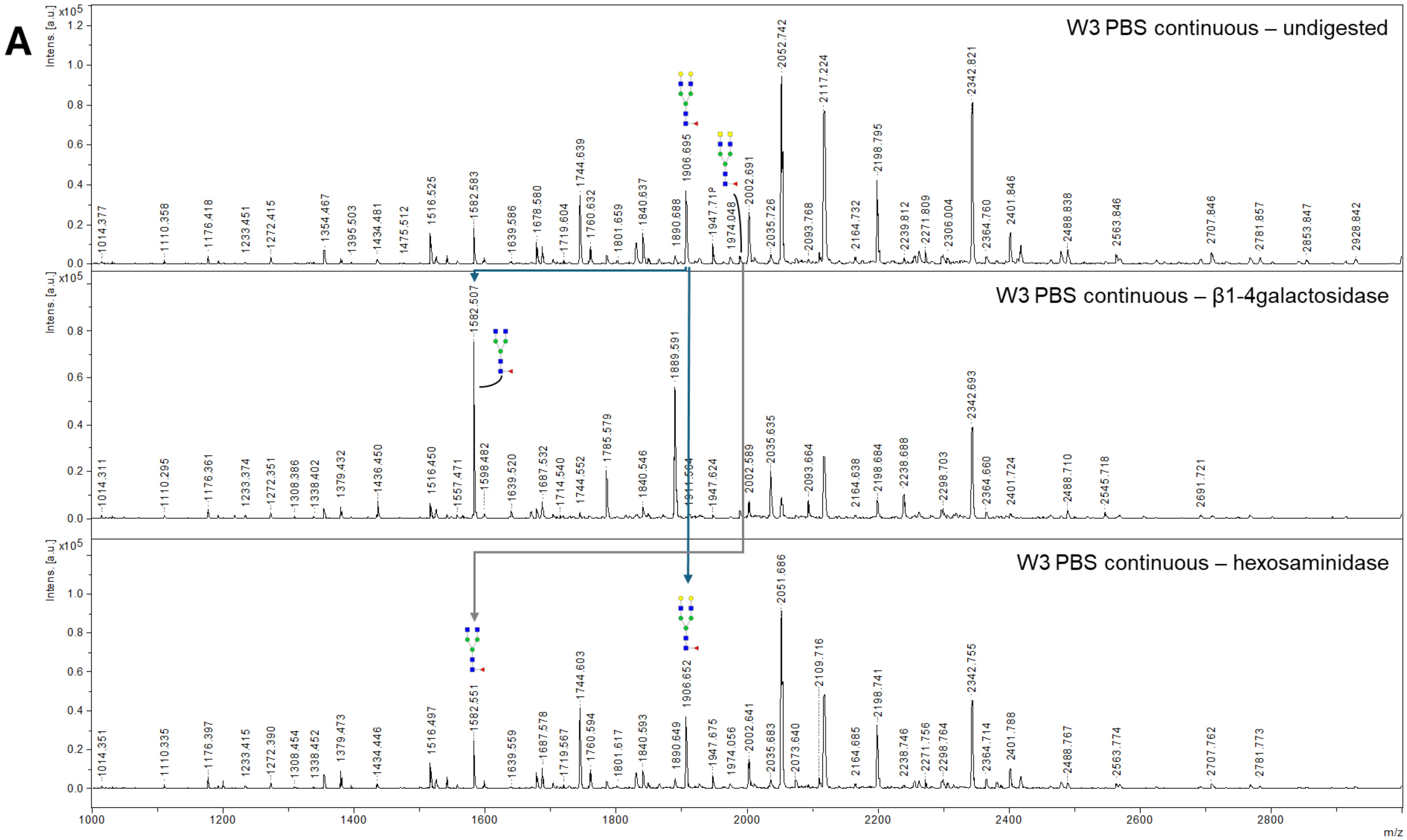

A
W3 PBS continuous – undigested
W3 PBS continuous – β1-4galactosidase
W3 PBS continuous – hexosaminidase
Intens. [a.u.]
x10^5
m/z

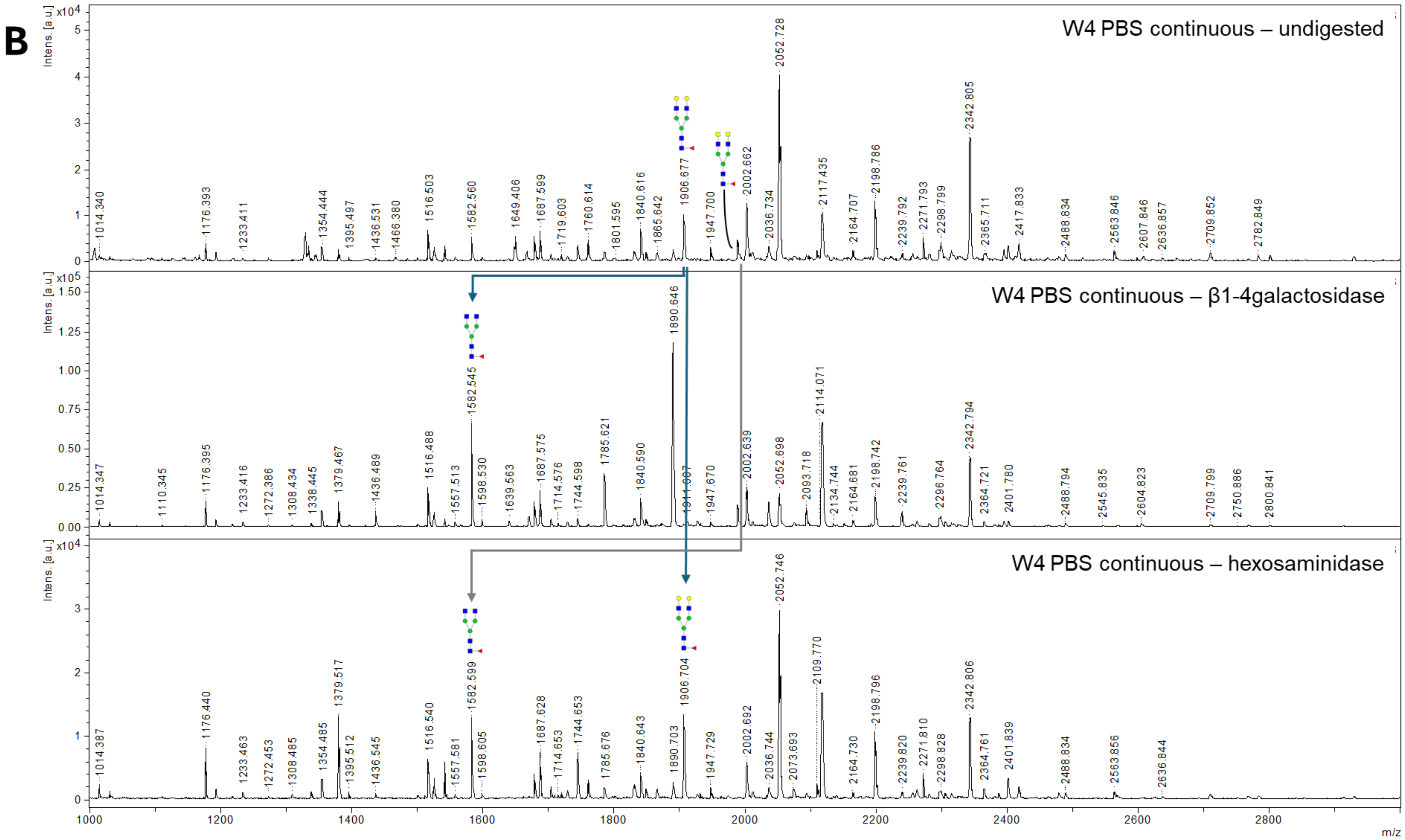

B
W4 PBS continuous – undigested
Intens. [a.u.]
x10^4
1014.340
1176.393
1233.411
1354.444
1395.497
1436.531
1466.380
1516.503
1582.560
1649.406
1687.599
1719.603
1760.614
1801.595
1840.616
1865.642
1906.677
1947.700
2002.662
2036.734
2052.728
2117.435
2164.707
2198.786
2239.792
2271.793
2298.799
2342.805
2365.711
2417.833
2488.834
2563.846
2607.846
2636.857
2709.852
2782.849
W4 PBS continuous – β1-4galactosidase
Intens. [a.u.]
x10^5
1014.347
1110.345
1176.395
1233.416
1272.386
1308.434
1338.445
1379.467
1436.489
1516.488
1557.513
1582.545
1598.530
1639.563
1687.575
1714.576
1744.598
1785.621
1840.590
1890.646
1947.670
2002.639
2052.698
2093.718
2114.071
2134.744
2164.681
2198.742
2239.761
2296.764
2342.794
2364.721
2401.780
2488.794
2545.835
2604.823
2709.799
2750.886
2800.841
W4 PBS continuous – hexosaminidase
Intens. [a.u.]
x10^4
1014.387
1176.440
1233.463
1272.453
1308.485
1354.485
1379.517
1395.512
1436.545
1516.540
1557.581
1582.599
1598.605
1687.628
1714.653
1744.653
1785.676
1840.643
1890.703
1906.704
1947.729
2002.692
2036.744
2052.746
2073.693
2109.770
2164.730
2198.796
2239.820
2271.810
2298.828
2342.806
2364.761
2401.839
2488.834
2563.856
2636.844
1000
1200
1400
1600
1800
2000
2200
2400
2600
2800
m/z

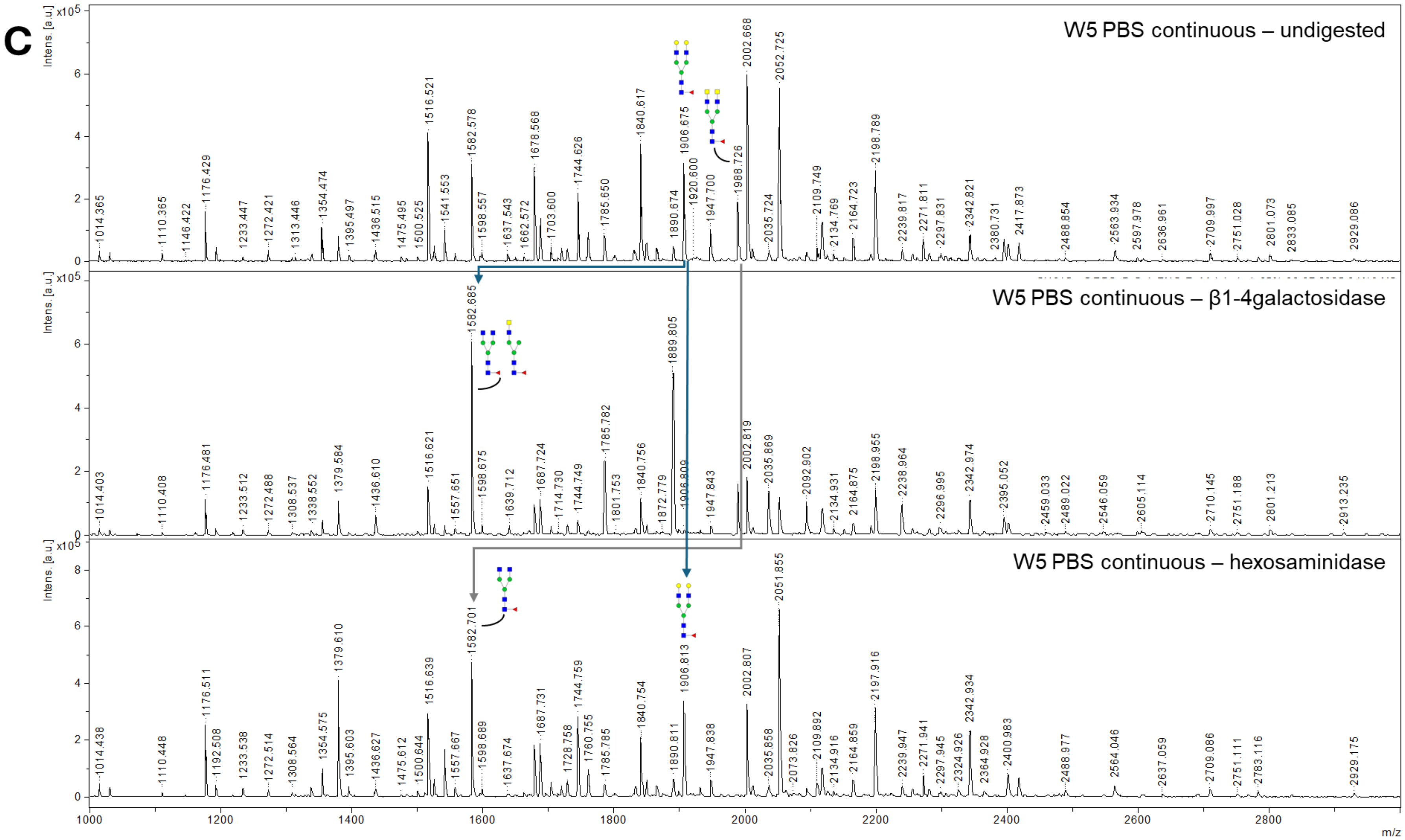
C
W5 PBS continuous – undigested
W5 PBS continuous – β1-4galactosidase
W5 PBS continuous – hexosaminidase
Intens. [a.u.]
x10^5
m/z
1014.365
1110.365
1146.422
1176.429
1233.447
1272.421
1313.446
1354.474
1395.497
1436.515
1475.495
1500.525
1516.521
1541.553
1582.578
1598.557
1637.543
1662.572
1678.568
1703.600
1744.626
1785.650
1840.617
1890.674
1906.675
1920.600
1947.700
1988.726
2002.668
2035.724
2052.725
2109.749
2134.769
2164.723
2198.789
2239.817
2271.811
2297.831
2342.821
2380.731
2417.873
2488.854
2563.934
2597.978
2636.961
2709.997
2751.028
2801.073
2833.085
2929.086
1014.403
1110.408
1176.481
1233.512
1272.488
1308.537
1338.552
1379.584
1436.610
1516.621
1557.651
1582.685
1598.675
1639.712
1687.724
1714.730
1744.749
1785.782
1801.753
1840.756
1872.779
1889.805
1906.809
1947.843
2002.819
2035.869
2092.902
2134.931
2164.875
2198.955
2238.964
2296.995
2342.974
2395.052
2459.033
2489.022
2546.059
2605.114
2710.145
2751.188
2801.213
2913.235
1014.438
1110.448
1176.511
1192.508
1233.538
1272.514
1308.564
1354.575
1379.610
1395.603
1436.627
1475.612
1500.644
1516.639
1557.667
1582.701
1598.689
1637.674
1687.731
1728.758
1744.759
1760.755
1785.785
1840.754
1890.811
1906.813
1947.838
2002.807
2035.858
2051.855
2073.826
2109.892
2134.916
2164.859
2197.916
2239.947
2271.941
2297.945
2324.926
2342.934
2364.928
2400.983
2488.977
2564.046
2637.059
2709.086
2751.111
2783.116
2929.175
1000
1200
1400
1600
1800
2000
2200
2400
2600
2800

D

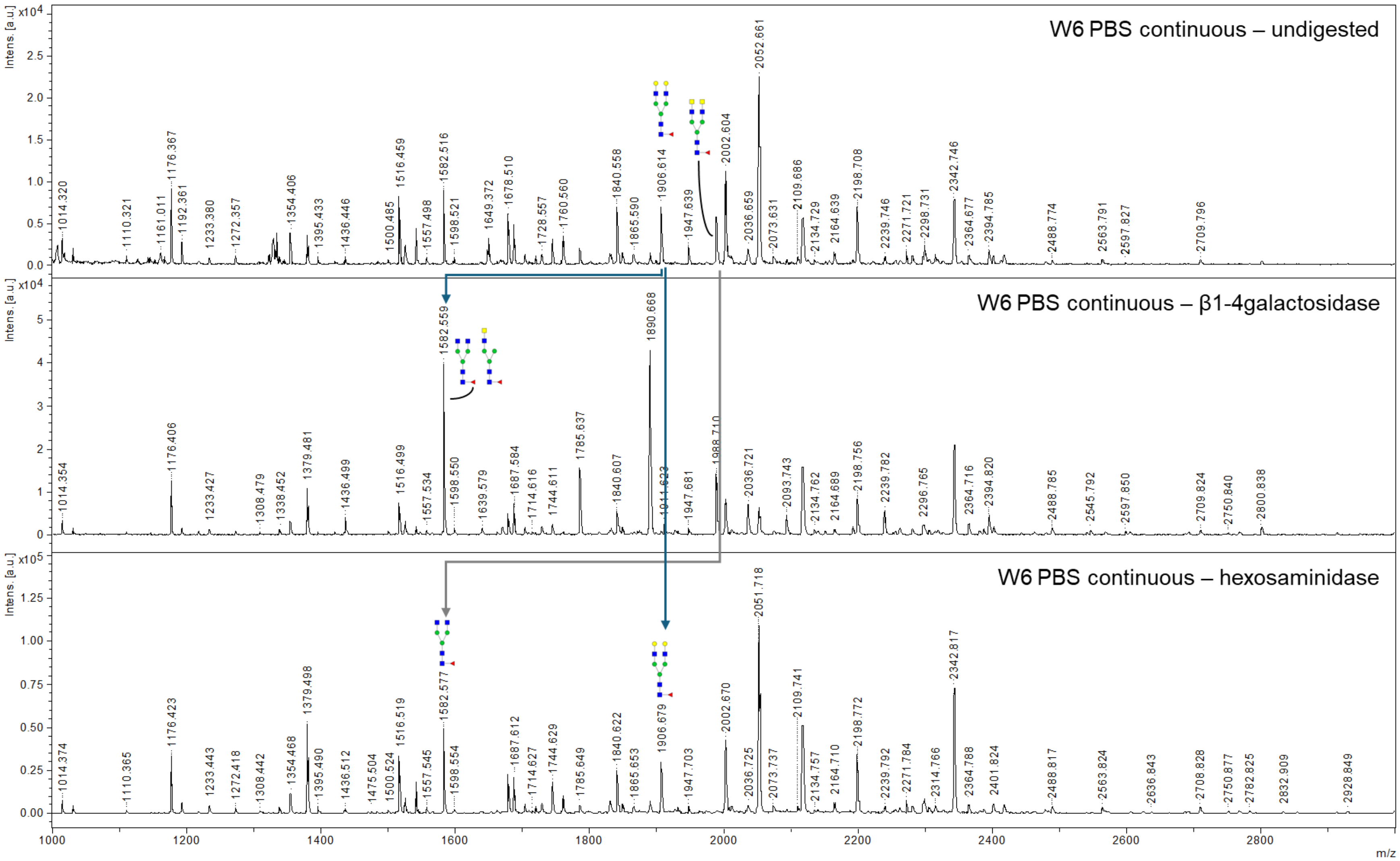
W6 PBS continuous – undigested
Intens. [a.u.]
x10^4
1014.320
1110.321
1161.011
1176.367
1192.361
1233.380
1272.357
1354.406
1395.433
1436.446
1500.485
1516.459
1557.498
1582.516
1598.521
1649.372
1678.510
1728.557
1760.560
1840.558
1865.590
1906.614
1947.639
2002.604
2036.659
2052.661
2073.631
2109.686
2134.729
2164.639
2198.708
2239.746
2271.721
2298.731
2342.746
2364.677
2394.785
2488.774
2563.791
2597.827
2709.796
W6 PBS continuous – β1-4galactosidase
x10^4
1014.354
1176.406
1233.427
1308.479
1338.452
1379.481
1436.499
1516.499
1557.534
1582.559
1598.550
1639.579
1687.584
1714.616
1744.611
1785.637
1840.607
1890.668
1911.622
1947.681
1988.710
2036.721
2093.743
2134.762
2164.689
2198.756
2239.782
2296.765
2364.716
2394.820
2488.785
2545.792
2597.850
2709.824
2750.840
2800.838
W6 PBS continuous – hexosaminidase
x10^5
1014.374
1110.365
1176.423
1233.443
1272.418
1308.442
1354.468
1379.498
1395.490
1436.512
1475.504
1500.524
1516.519
1557.545
1582.577
1598.554
1687.612
1714.627
1744.629
1785.649
1840.622
1865.653
1906.679
1947.703
2002.670
2036.725
2051.718
2073.737
2109.741
2134.757
2164.710
2198.772
2239.792
2271.784
2314.766
2342.817
2364.788
2401.824
2488.817
2563.824
2636.843
2708.828
2750.877
2782.825
2832.909
2928.849
m/z

E

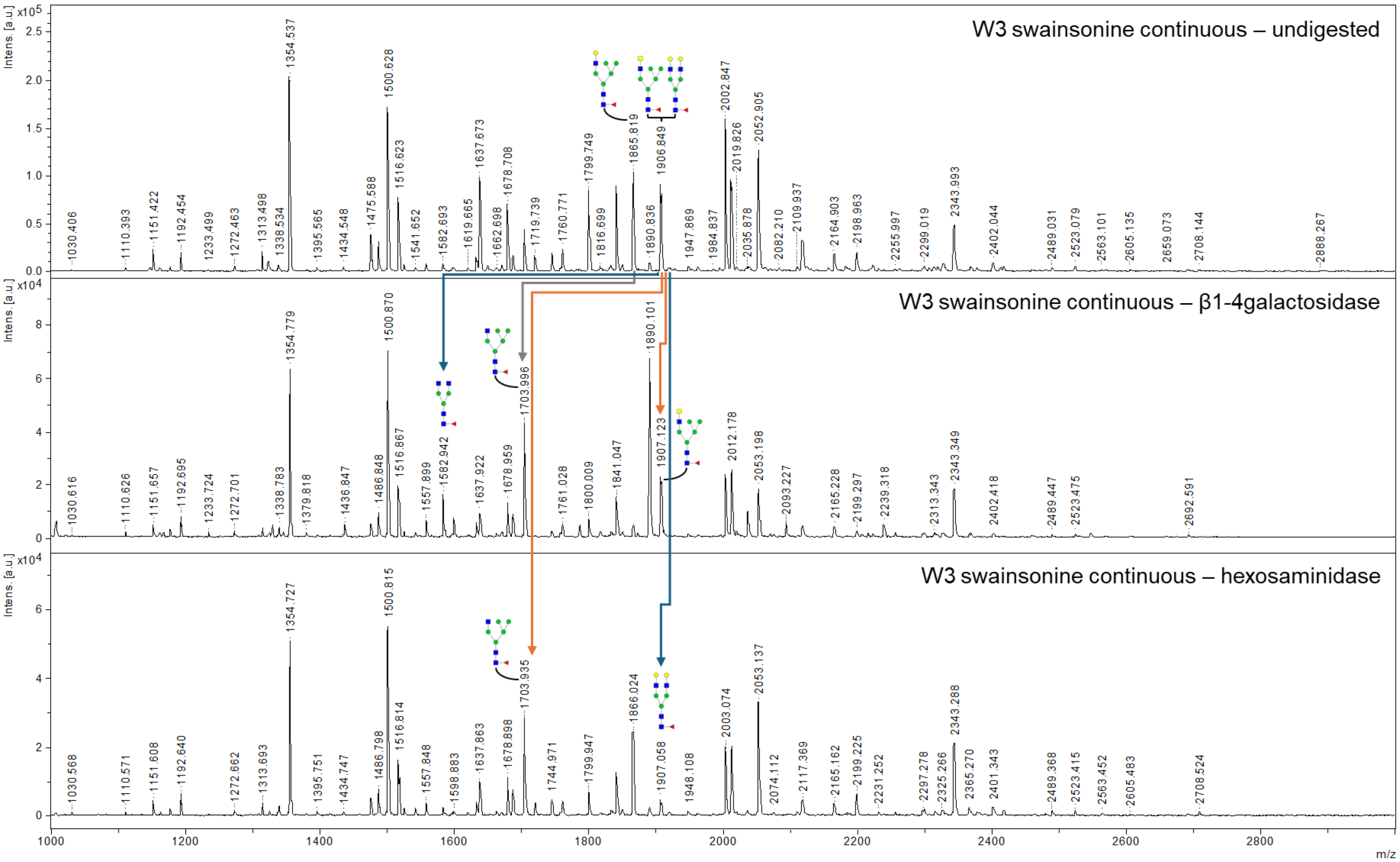

W3 swainsonine continuous – undigested
W3 swainsonine continuous – β1-4galactosidase
W3 swainsonine continuous – hexosaminidase
Intens. [a.u.]
x10^5
x10^4
m/z
1000
1200
1400
1600
1800
2000
2200
2400
2600
2800
1030.406
1110.393
1151.422
1192.454
1233.499
1272.463
1313.498
1338.534
1354.537
1395.565
1434.548
1475.588
1500.628
1516.623
1541.652
1582.693
1619.665
1637.673
1662.698
1678.708
1719.739
1760.771
1799.749
1816.699
1865.819
1890.836
1906.849
1947.869
1984.837
2002.847
2019.826
2035.878
2052.905
2082.210
2109.937
2164.903
2198.963
2255.997
2299.019
2343.993
2402.044
2489.031
2523.079
2563.101
2605.135
2659.073
2708.144
2888.267
1030.616
1110.626
1151.657
1192.695
1233.724
1272.701
1338.783
1354.779
1379.818
1436.847
1486.848
1500.870
1516.867
1557.899
1582.942
1637.922
1678.959
1703.996
1761.028
1800.009
1841.047
1890.101
1907.123
2012.178
2053.198
2093.227
2165.228
2199.297
2239.318
2313.343
2343.349
2402.418
2489.447
2523.475
2692.591
1030.568
1110.571
1151.608
1192.640
1272.662
1313.693
1354.727
1395.751
1434.747
1486.798
1500.815
1516.814
1557.848
1598.883
1637.863
1678.898
1703.935
1744.971
1799.947
1866.024
1907.058
1948.108
2003.074
2053.137
2074.112
2117.369
2165.162
2199.225
2231.252
2297.278
2325.266
2343.288
2365.270
2401.343
2489.368
2523.415
2563.452
2605.483
2708.524

F

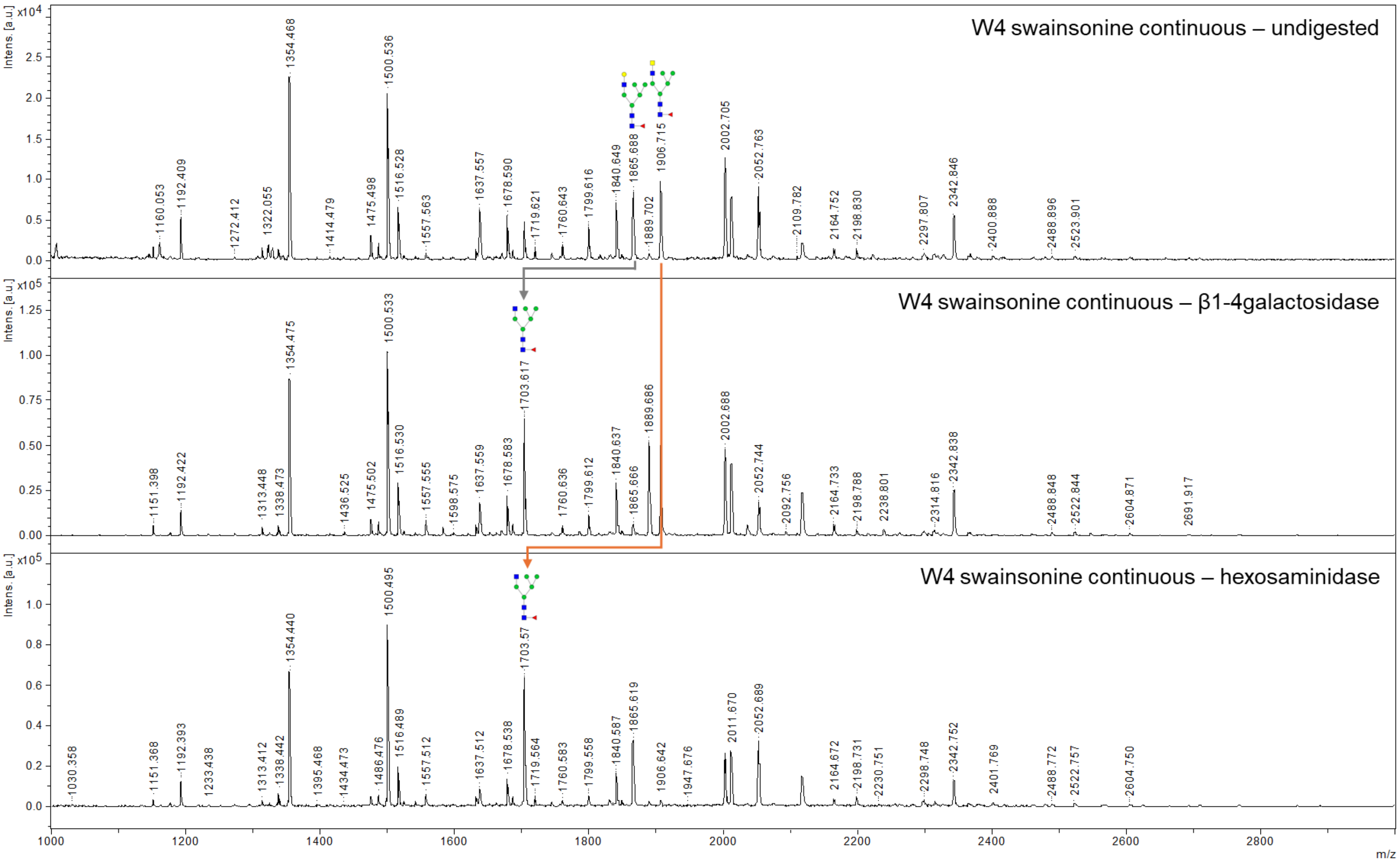
W4 swainsonine continuous – undigested
W4 swainsonine continuous – β1-4galactosidase
W4 swainsonine continuous – hexosaminidase
Intens. [a.u.]
m/z

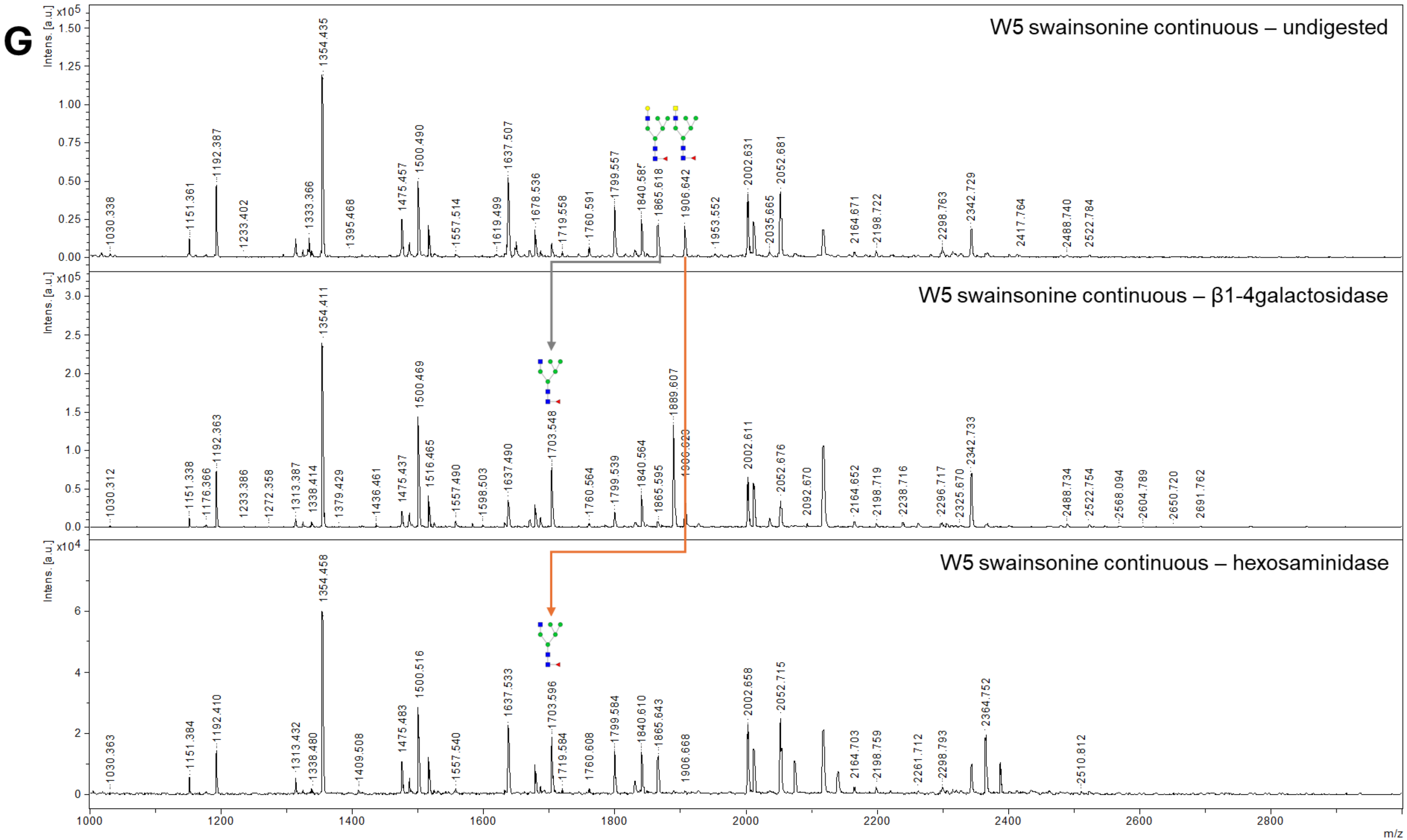
G
W5 swainsonine continuous – undigested
W5 swainsonine continuous – β1-4galactosidase
W5 swainsonine continuous – hexosaminidase
Intens. [a.u.]
x10^5
x10^4
m/z
1354.435
1192.387
1500.490
1637.507
1865.618
1906.642
2002.631
2052.681
2342.729
1354.411
1500.469
1703.548
1889.607
1354.458
1500.516
1703.596
2364.752

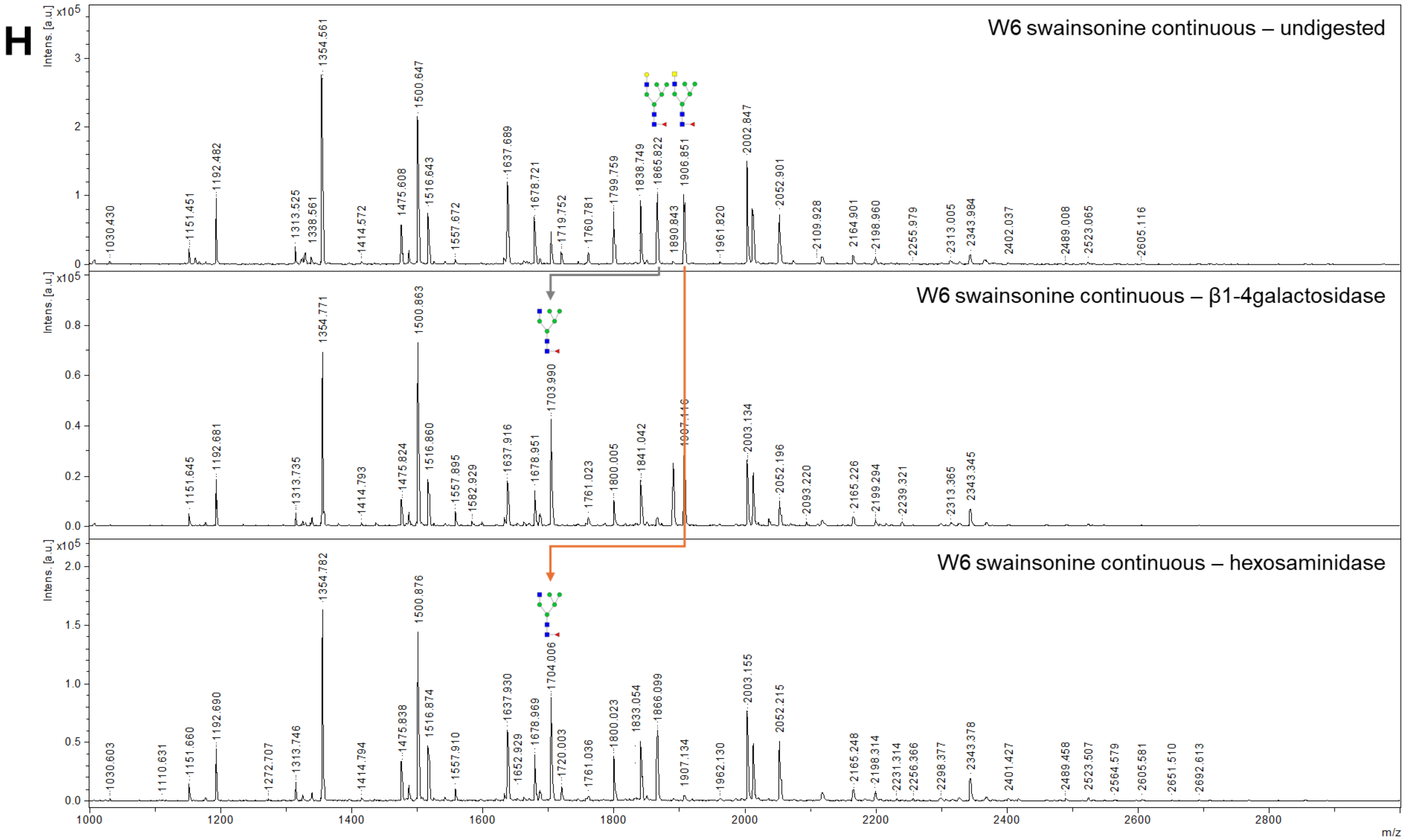

H
W6 swainsonine continuous – undigested
W6 swainsonine continuous – β1-4galactosidase
W6 swainsonine continuous – hexosaminidase
Intens. [a.u.]
x10^5
m/z

I

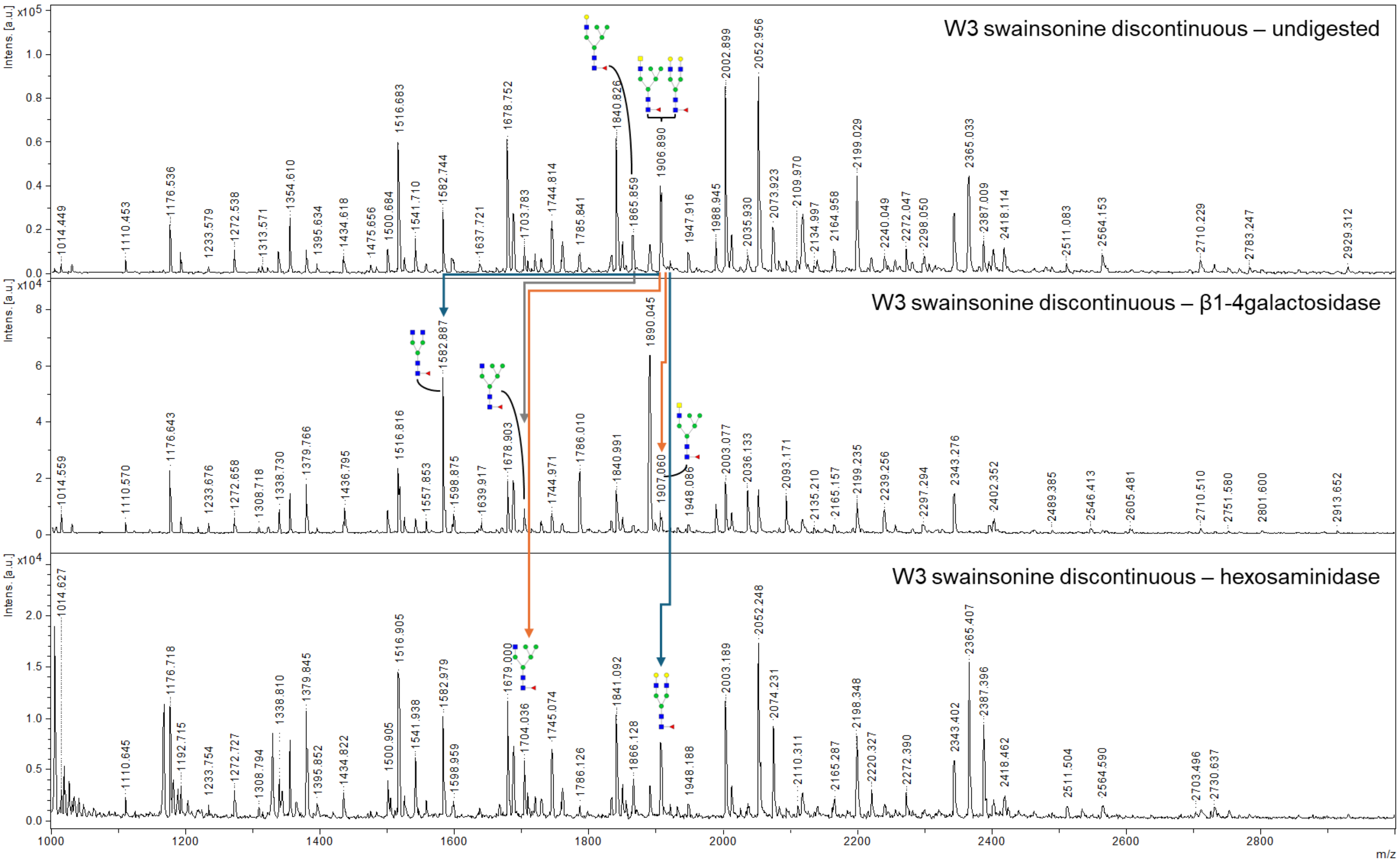
W3 swainsonine discontinuous – undigested
W3 swainsonine discontinuous – β1-4galactosidase
W3 swainsonine discontinuous – hexosaminidase
Intens. [a.u.]
m/z

J

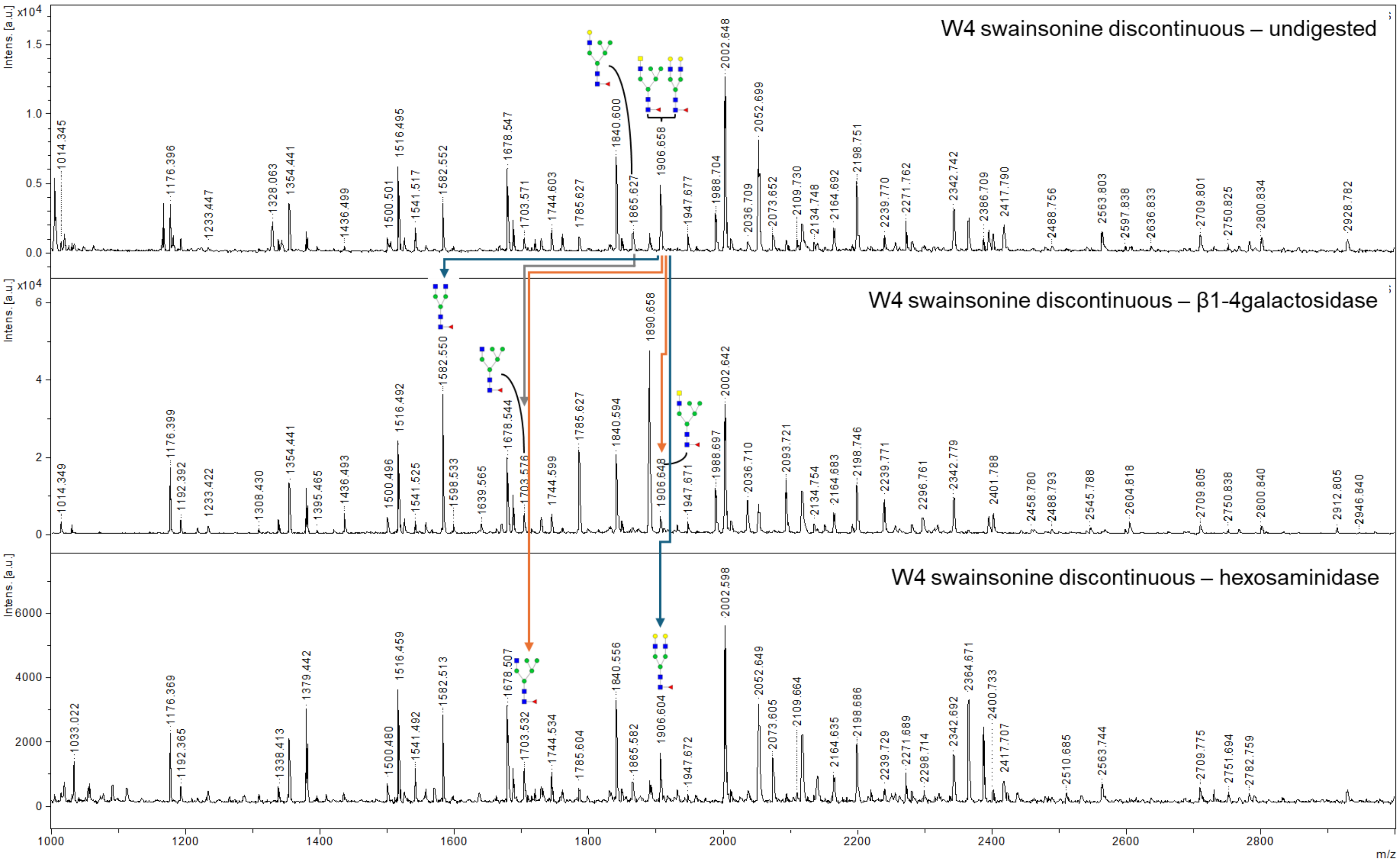
W4 swainsonine discontinuous – undigested
W4 swainsonine discontinuous – β1-4galactosidase
W4 swainsonine discontinuous – hexosaminidase
Intens. [a.u.]
m/z

K

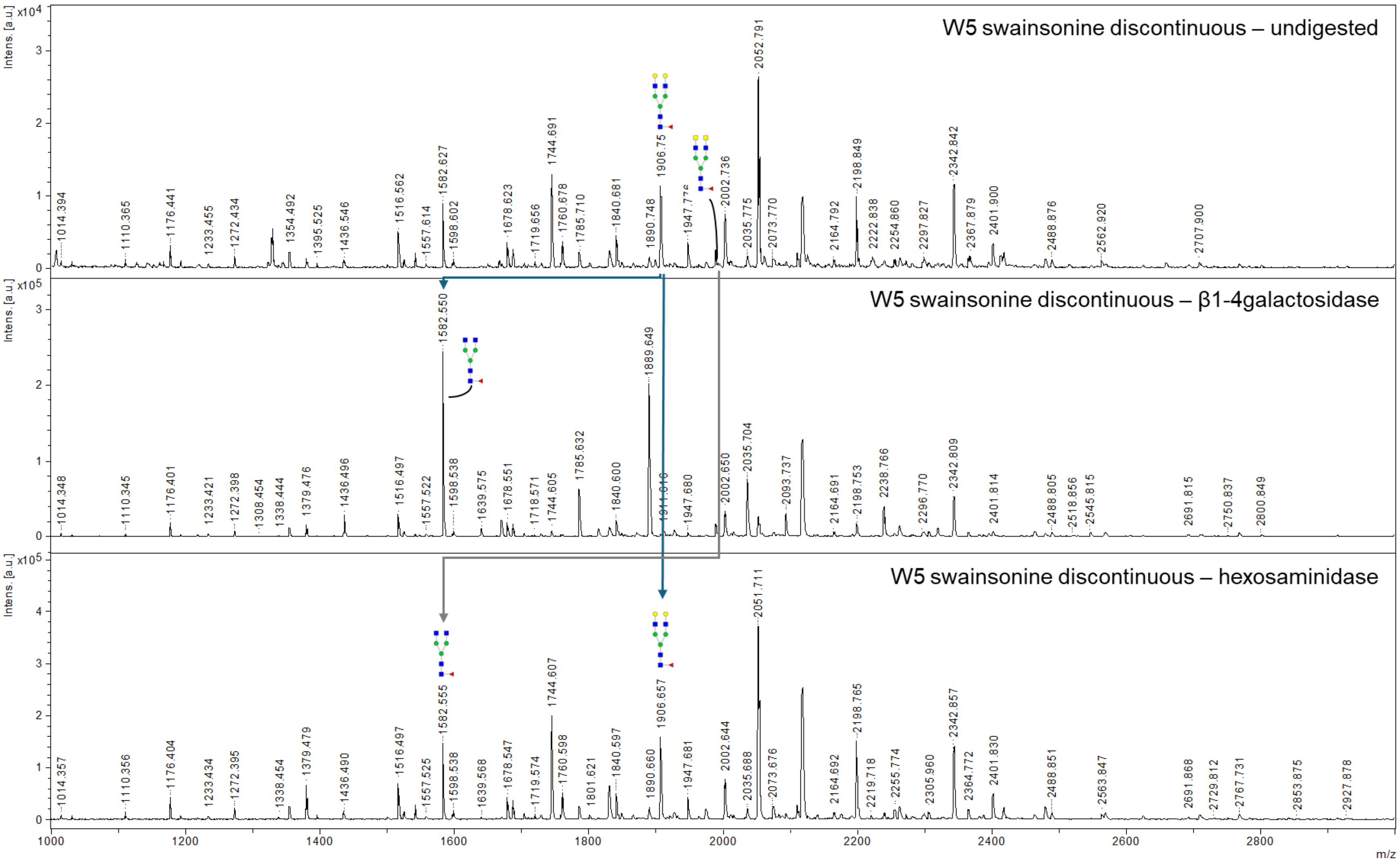
W5 swainsonine discontinuous – undigested
W5 swainsonine discontinuous – β1-4galactosidase
W5 swainsonine discontinuous – hexosaminidase
Intens. [a.u.]
m/z

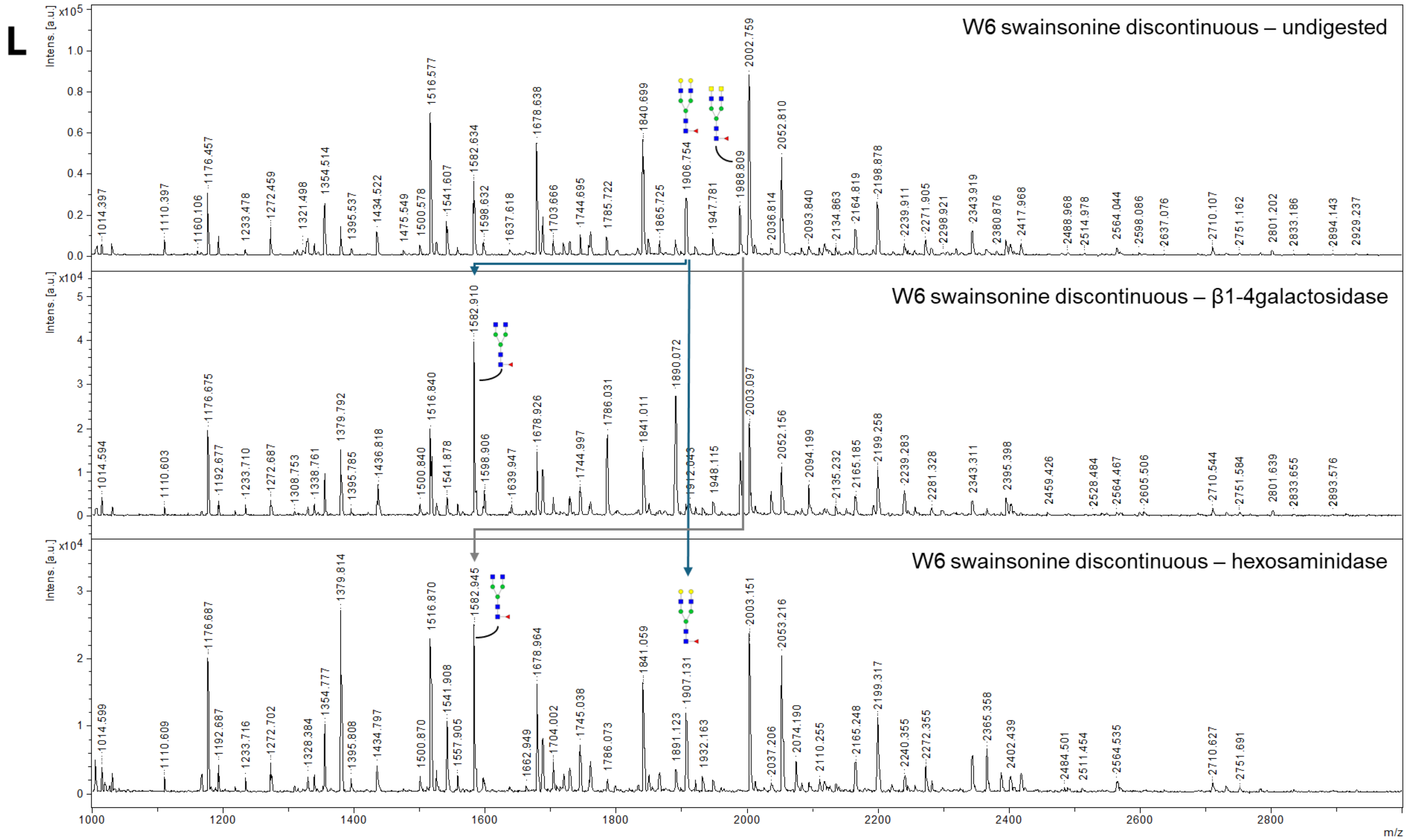

Supplementary figure 4 MALDI-TOF mass spectra of N-glycans before and after digestion with hexosaminidase and β1-4 galactosidase, derived from W3-W6 PBS-control schistosomula (A-D), schistosomula with continued swainsonine treatment (E-H) and discontinued swainsonine treatment (I-L). Green circle = mannose, yellow circle = galactose, blue circle = glucose, yellow square = N-acetylgalactosamine, blue square = N-acetylglucosamine, red triangle = fucose. Glycan images were generated in GlycoWorkBench [46].

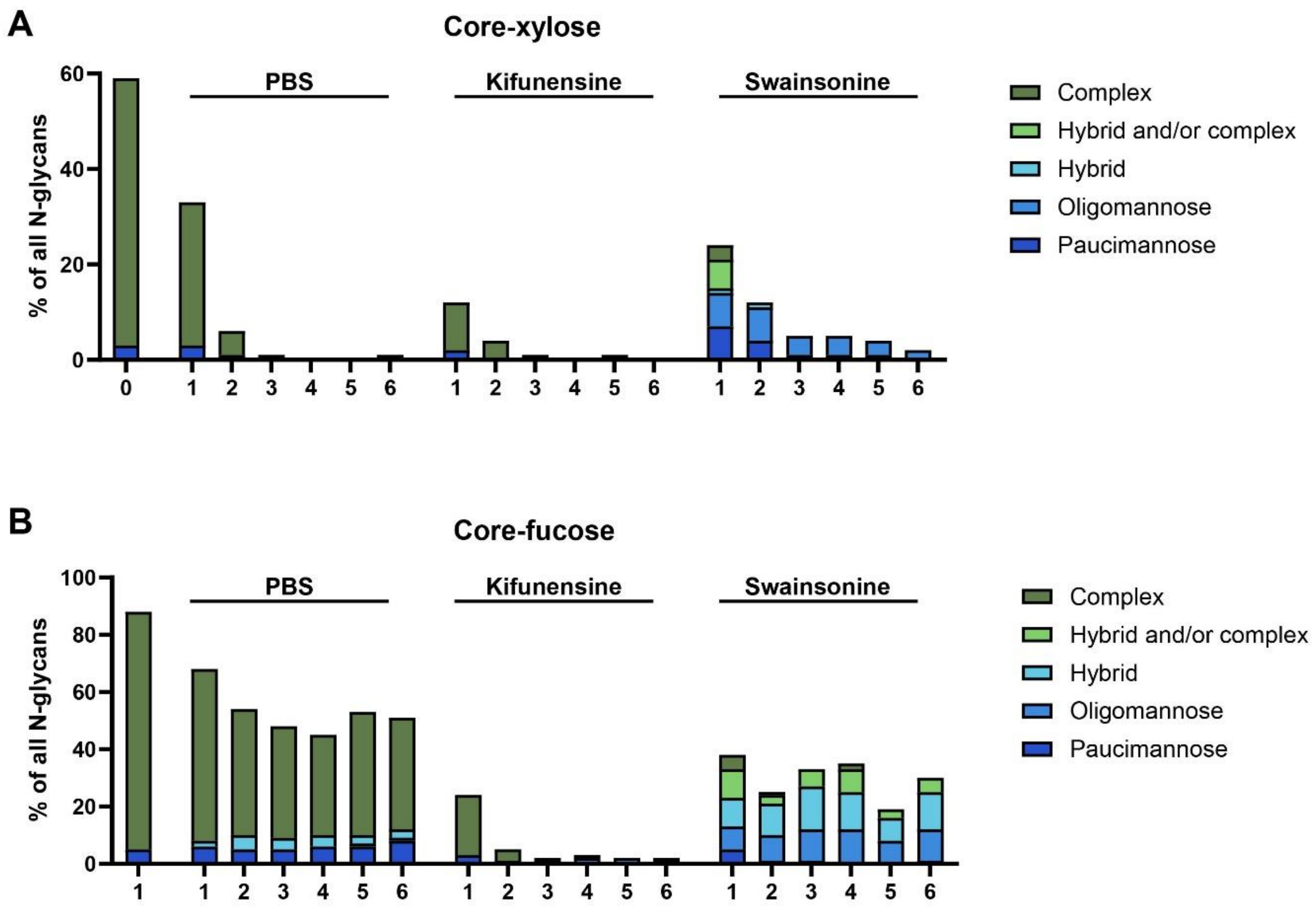

Supplementary figure 5 Presence of core-xylose and core-fucose in separate N-glycan categories Presence of core-xylose (A) and core-fucose (B) depicted for every N-glycan category for PBS-control, kifunensine- and swainsonine-treated schistosomula. The median of three biological replicates is shown.

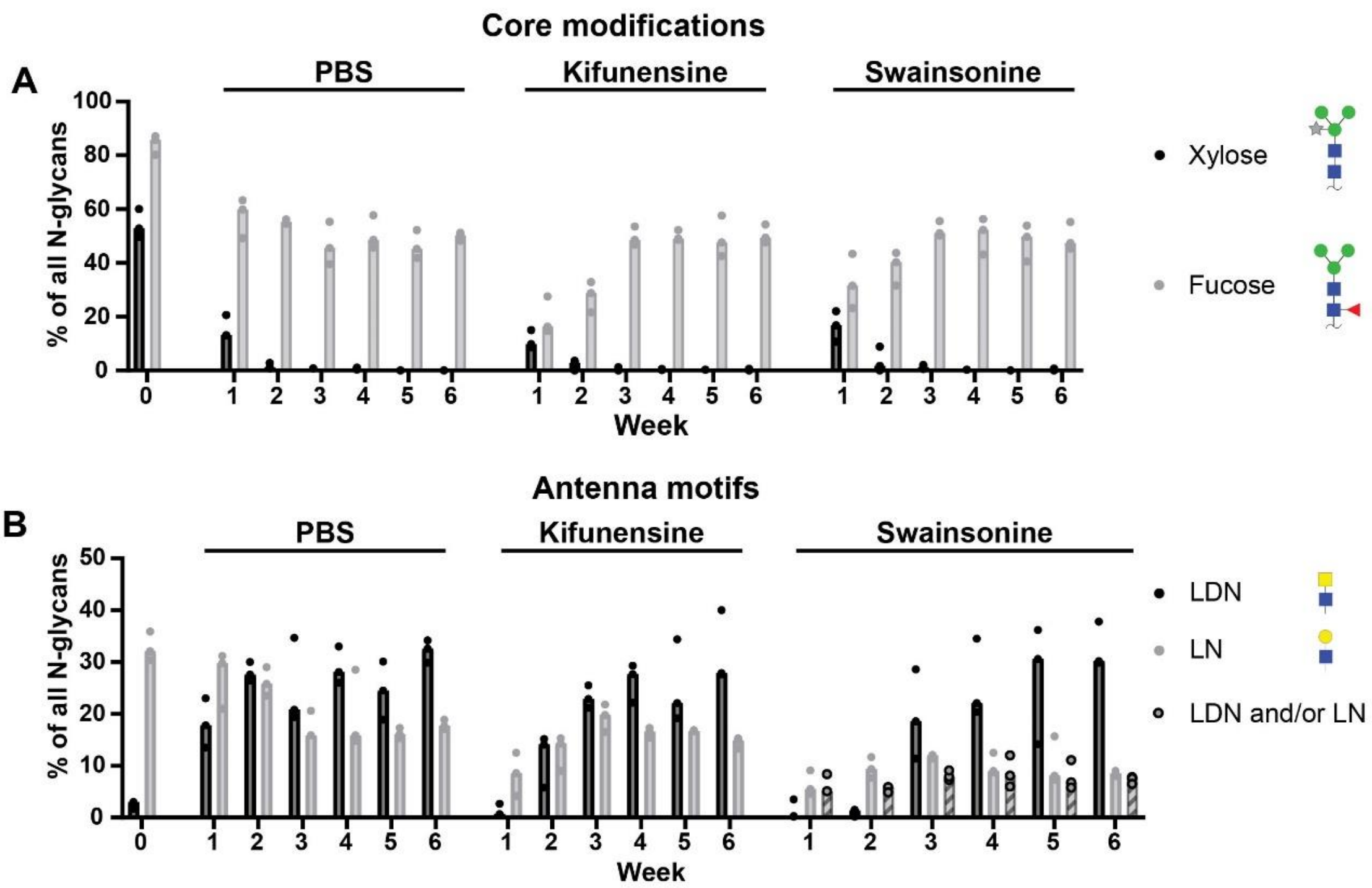

Supplementary figure 6 Presence of N-glycan motifs of freshly transformed (W0) as well as discontinued PBS-control, kifunensine- and swainsonine-treated schistosomula
Presence of the N-glycan motifs core-xylose and core-fucose (A), and LacdiNAc (LDN; GalNAcβ1-4GlcNAc) and LacNAc (LN; Galβ1–4GlcNAc) (B), are shown for W0 schistosomula and W1 to W6 of discontinued PBS-control, kifunensine- and swainsonine-treated schistosomula. The median of three biological replicates is shown with dots representing individual biological replicates. Green circle = mannose, yellow circle = galactose, yellow square = N-acetylgalactosamine, blue square = N-acetylglucosamine, red triangle = fucose, grey star = xylose.

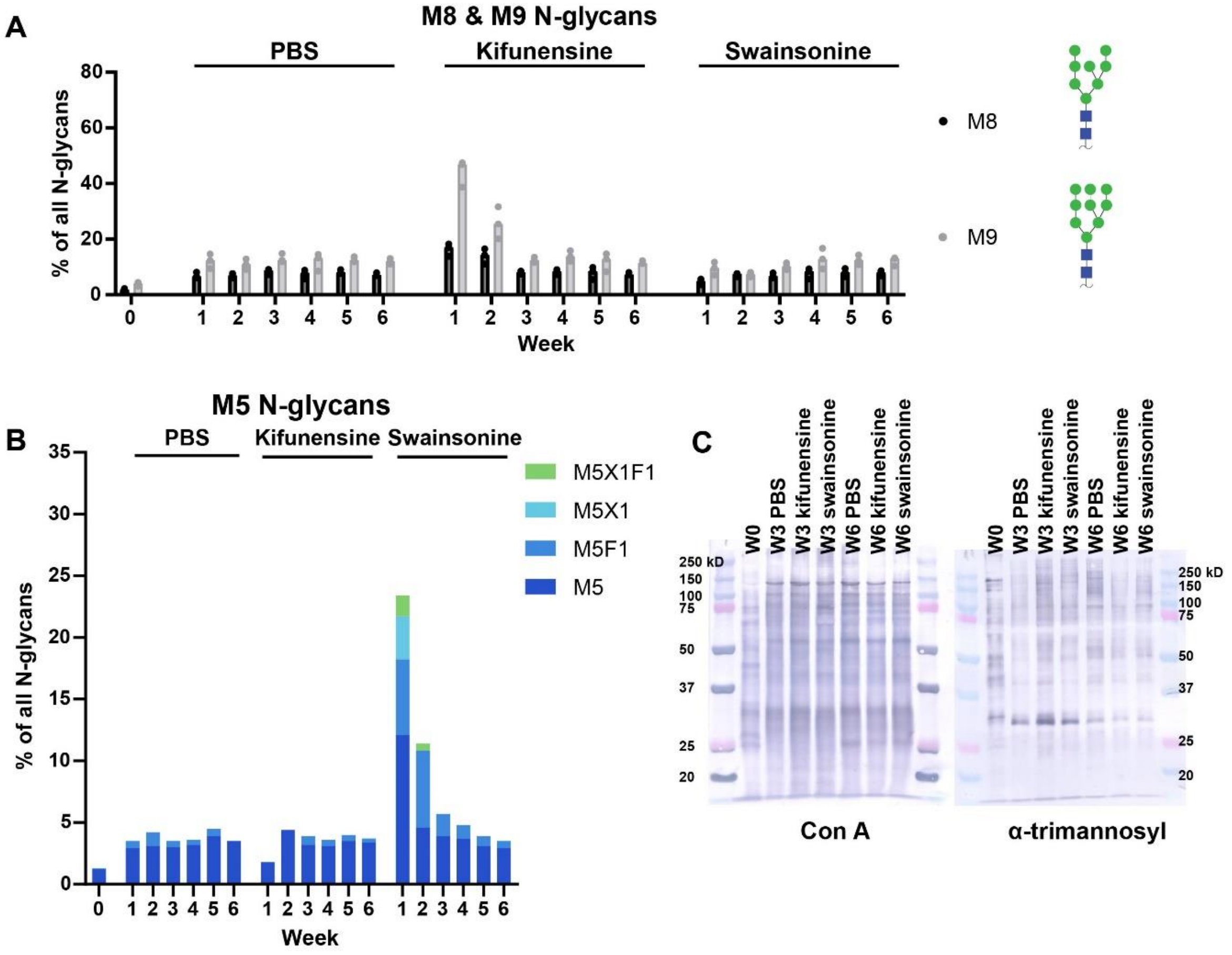

Supplementary figure 7 Presence of α-mannosidase inhibition-specific markers in freshly transformed (W0) as well as discontinued PBS-control, kifunensine- and swainsonine-treated schistosomula

The kifunensine-specific markers M9 (Man9GlcNAc2) and M8 (Man8GlcNAc2) (A), and swainsonine-specific marker (core-modified) M5 (Man5GlcNAc2) (B) are shown for W0 schistosomula and discontinued W1 to W6 of PBS-control, kifunensine- and swainsonine-treated schistosomula. The median of three biological replicates is shown with dots representing individual biological replicates (A-B). Lectin and Western blots containing protein extracts of W0, W3 and W6 schistosomula were incubated with Con A, a lectin with affinity for mostly high mannose (M7-M9) N-glycans, and an antibody for trimannosyl (Manα1-3(Manα1-6)Man). A representative blot of two biological replicates is shown (C). Green circle = mannose, blue square = N-acetylglucosamine.

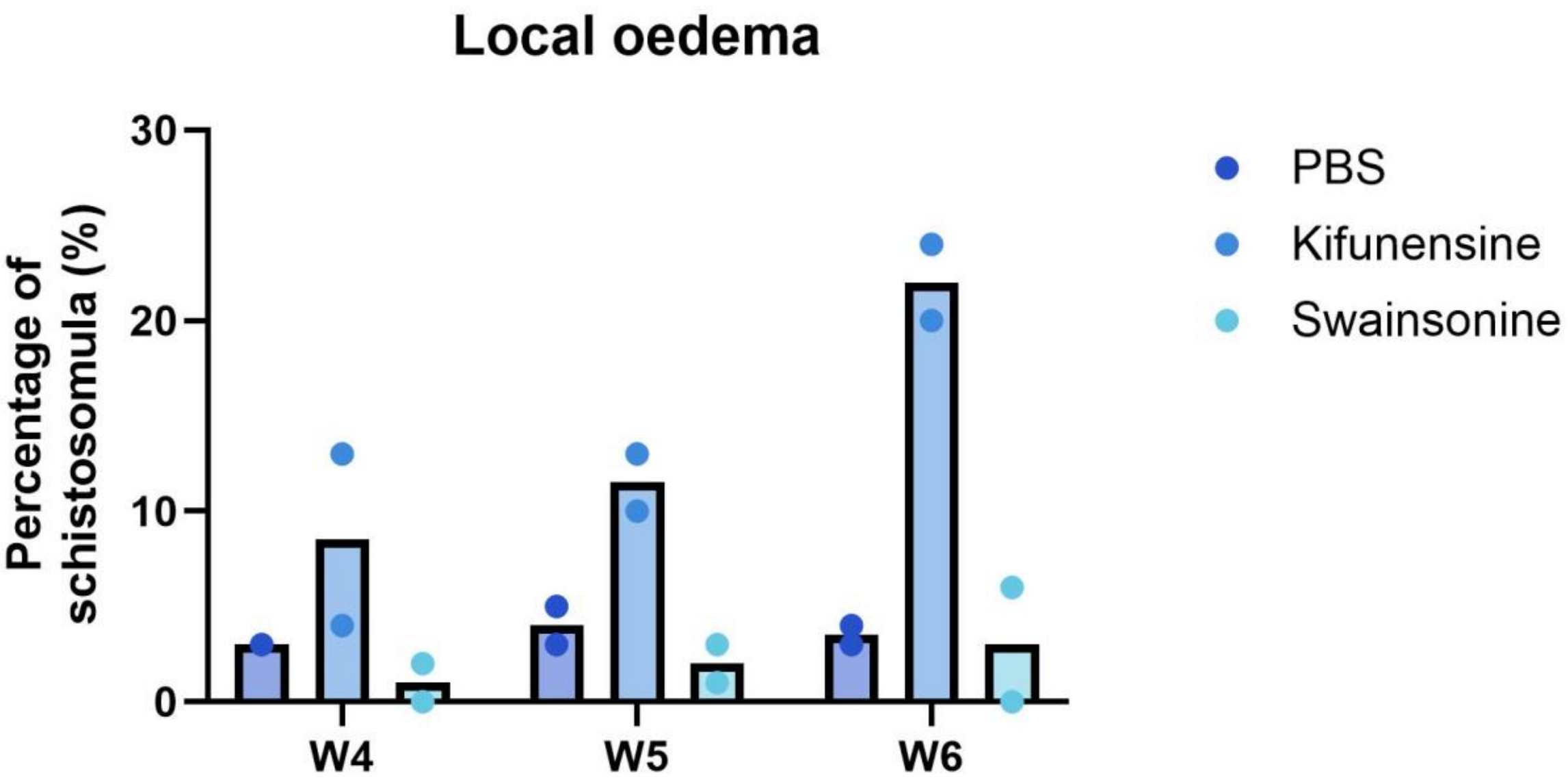

Supplementary figure 8 Local oedema observed in schistosomula with and without α-mannosidase inhibition through brightfield microscopy
The median percentage of local oedema at W4, W5 and W6 schistosomula cultured with PBS as control, kifunensine and swainsonine is shown. One biological replicate is shown with each dot representing the quantification by an independent, blinded researcher.
